# Divergent Clonal Evolution and Early Dissemination Promote Genetic Heterogeneity of Metastases in Castration-Resistant Prostate Cancer

**DOI:** 10.1158/0008-5472.CAN-24-3687

**Published:** 2025-08-18

**Authors:** Noshad Hosseini, Rahul Mannan, Ryan J. Rebernick, Fengyun Su, Rui Wang, Xuhong Cao, Ana Lako, Dattatreya Mellacheruvu, Jing Hu, Joshi J. Alumkal, Zachery R. Reichert, Rohit Malik, Rohit Mehra, Arul M. Chinnaiyan, Marcin P. Cieslik

**Affiliations:** 1 Gilbert S. Omenn Department of Computational Medicine and Bioinformatics, University of Michigan, Ann Arbor, Michigan.; 2 Michigan Center for Translational Pathology, University of Michigan, Ann Arbor, Michigan.; 3 Department of Pathology, University of Michigan, Ann Arbor, Michigan.; 4 Bristol Myers Squibb, Lawrenceville, New Jersey.; 5 Department of Internal Medicine, University of Michigan, Ann Arbor, Michigan.; 6 Rogel Cancer Center, University of Michigan, Ann Arbor, Michigan.; 7 Howard Hughes Medical Institute, Ann Arbor, Michigan.; 8 Division of Diagnostic Genetics and Genomics, University of Michigan, Ann Arbor, Michigan.

## Abstract

Lethal prostate cancer has passed through at least two evolutionary bottlenecks: acquisition of metastatic potential and development of castration resistance. A better understanding of how this affects genetic heterogeneity across metastatic sites is needed to develop strategies to block metastatic spread and to overcome resistance. By leveraging deep whole-exome sequencing of 93 tumors from 26 patients, we examined patterns of metastatic dissemination and clonal evolution of prostate cancer. Phylogenetic reconstruction and mathematical modeling enabled quantification of the number of mutations and clones in the cancer ecosystem and characterization of each patient’s disease as an evolutionary process. Although mutations of the earliest pathogenic genetic drivers of prostate cancer were truncal, most other likely passenger mutations, copy-number alterations, and clones arose after the cancer had spread and were confined to individual metastatic sites because of polyclonal and polyphyletic seeding. Single-tissue sequencing tended to overestimate mutation clonality and, apart from truncal drivers, underestimate mutation rates for both individual patients and cohorts. This study highlights the independent evolution of metastatic lesions, which has implications for diagnostic and targeted therapy strategies.

**Significance:**

Genomic analysis of a multisite metastatic prostate cancer cohort reveals patterns of clonal heterogeneity, dissemination, and evolution, highlighting the need to evaluate multiple samples to fully characterize the clonal architecture of tumors.

*This article is part of a special series: Driving Cancer Discoveries with Computational Research, Data Science, and Machine Learning/AI* .

## Introduction

Every cancer harbors some degree of heterogeneity (1–3), which arises from genetic alterations in a subset of cells within a tumor (i.e., clones), resulting in functional and phenotypic differences among clones (1). Prostate cancer is known to be a notably genetically heterogeneous disease (4, 5), which presents challenges in diagnosis and treatment (6). Genetic heterogeneity across metastatic lesions can lead to resistance to treatment (2, 7) and limit the utility of diagnostic approaches, biomarker assays, and drug targets that rely on sequencing from a single site (8, 9). Underpinning this heterogeneity are the high clonal diversity (7) and multifocality (10) of metastatic disease and primary disease, respectively. For example, research by Lovf and colleagues (11) reports that within a prostatectomy, different foci frequently have distinct driver aberrations, with as many as 76% of prostate cancer foci sharing no mutations. Kim and colleagues (12) described that even in patients in whom there is a common ancestor among different foci, a single primary tumor biopsy can harbor as few as 20% of the mutations detected across all foci.

Although the genetic and histologic heterogeneity of primary and locoregional prostate cancer is well documented (4, 9–11, 13), most studies of metastatic castration-resistant prostate cancer (mCRPC) have relied primarily on a single sample from each patient, which underestimates the disease’s heterogeneity (4, 14). Landmark multisite sequencing studies of mCRPC have uncovered complex patterns and mechanisms of seeding (7, 15), phenotypic heterogeneity (16), and significant androgen receptor (*AR*) genomic complexity (7, 17), a striking finding considering that lethal prostate cancer has passed through at least two evolutionary bottlenecks: acquisition of metastatic potential and development of castration resistance. However, these studies are also limited by shallow sequencing depth, small number of patients, or narrow focus (7, 15, 17). The breadth of clonal heterogeneity within mCRPC is inadequately described, thus limiting our understanding of the factors related to clonal selection and genetic drift. Understanding these factors may allow interventions to block the two most important events in the disease process: metastatic spread and resistance.

This study addresses this gap in knowledge by analyzing postmortem samples of a cohort of 26 patients with mCRPC with distant metastases enrolled in our rapid autopsy program (Michigan Legacy Tissue Program). Leveraging high-depth sequencing and mathematical modeling, we characterized the extent of mutational, copy-number (CN), and clonal heterogeneity. We quantitatively evaluate the impact of intertumor genetic heterogeneity on patient mutation detection rates. Furthermore, to uncover pathways to metastasis between different tumor sites, we explored phylogenies of tumor clonal evolution, patterns of seeding and migration, and evolutionary timing of genetic alterations. Lastly, we characterized an instructive case of mCRPC with distinct differentiation phenotypes, early clonal branching, and metastatic dissemination. Altogether, this study highlights the independent evolution of metastatic lesions and its implications for diagnostic and targeted therapy strategies.

## Materials and Methods

### Sample collection

Frozen tissue from all tumors and matched normal specimens were obtained from the Michigan Legacy Tissue Program. The rapid autopsy protocol has been described previously (18, 19) and was conducted with the approval of the University of Michigan Institutional Review Board in accordance with the US Common Rule (45 CFR 46). Written informed consent was obtained from patients or by next of kin. Briefly, patients with diagnosed castration-resistant prostate cancer were referred to Medical Oncology Service of the Comprehensive Cancer Center at the University of Michigan Hospitals. According to patient consent, autopsies were performed on these patients. These autopsies have been referred to as “warm” or recent autopsies because of the short time interval between patient death and starting the necropsy. According to the rapid autopsy protocol, if the prostate was in place, the prostate, urinary bladder, and pelvic lymph nodes were resected in a manner similar to that of a cystoprostatectomy. In patients with previous prostatectomy, the remaining content of the pelvic was removed. This included an evaluation of the pelvic lymph nodes for disease. Next, rapid processing of fresh tissue samples began, tissues procured at the time of autopsy were placed in optimal cutting temperature medium (Sakura Finetek USA) or formalin for frozen or permanent histologic sections (20), respectively, and frozen tissue was stored at −70°C. Autopsies of patients in our cohort was captured in the timeframe between the year 1997 (WA_4) and 2020 (WA_74).

### Preparation of whole-exome and RNA sequencing libraries

Exome sequencing and exome-capture RNA sequencing (RNA-seq) were performed using standard protocols in our Clinical Laboratory Improvement Amendments–compliant sequencing laboratory (21). Briefly, tumor DNA and total RNA were purified from the same sample using the AllPrep DNA/RNA/miRNA kit (Qiagen). Matched normal genomic DNA from normal tissue was isolated using the DNeasy Blood & Tissue kit (QIAGEN). The exome-capture transcriptome platform was used for RNA-seq. Exome libraries of matched pairs of tumor and normal DNA were prepared using the SureSelect Human All Exon V4 platform (Agilent) as previously described (22). All the samples were sequenced on a HiSeq 2500 or NovaSeq instrument (Illumina) in paired-end 150 bp mode. The primary base call files were converted into FASTQ sequence files using the bcl2fastq version 1.8.4 converter tool.

### Comprehensive genomics analysis pipeline

All genomic data processing and analysis have been performed using the Turnkey Precision Oncology (TPO) workflow (https://github.com/mctp/tpo), which implements standardized pipelines for the analysis of DNA sequencing and RNA-seq data, broadly following community best practices (23). Relevant algorithmic details are explained below, and a general overview of its functions has been provided in prior publications (24–26). Briefly, in our pipeline after the read grooming/quality control (QC), alignment, and deduplication of reads, we first infer somatic and germline variants separately (using dedicated somatic and/or germline variant callers). Then we use the alignment files (to calculate the coverage of each locus) and germline variants (to calculate the allelic imbalances) to calculate two values: the log coverage ratio (LR) and B-allele frequency (BAF). Next, using an established likelihood-based model, we input LR and BAF into our model to find the most likely purity and ploidy values. These two values need to be calculated together, as our likelihood model is dependent on both of them. Lastly, having purity, ploidy, LR, and BAFs, we calculated the absolute CN of each segment on the gene. Following this, it becomes possible to infer the multiplicity of each variant (germline or somatic).

### Read processing and data QC

BBMap’s bbduk (https://sourceforge.net/projects/bbmap/) was used to perform trimming of DNA sequencing paired-end reads. Next, the data were aligned to the GRCh38 reference using BWA-MEM (27). The Sentieon sort tool (bioRxiv 2017.05.12.115717v2) was also utilized to sort the reads in the BAM files. Further QC measures were extracted from the data according to GATK Best Practices.

### Somatic variant calling

For somatic variants, all tumor samples were matched with a normal tissue. TNscope (bioRxiv 2017.05.12.115717v2), an improved somatic caller based on GATK Mutect2, was used to call the somatic variants and calculate variant allele frequency (VAF) according to its probabilistic model, taking realignment and mapping ambiguity into account. The following settings were used in TNscope:

--max_fisher_pv_active 0.05 --min_tumor_allele_frac 0.0075 --min_init_tumor_lod 2.5 --assemble_mode 4 --trim_soft_clip --normal_contamination_frac 0.25 --prune_factor 4.

This setting was particularly selected to allow for variant calls with presented evidence in normal tissue and tumor tissue. Such an approach allowed us to later account for possible tumor-in-normal (TIN) contaminations (see Somatic variant calling QC). The called variants were annotated using the Ensembl Variant Effect Predictor (VEP; ref. 28) and vcfanno (29). All detected variants were further filtered (to retain somatic variants) according to a rule-based pipeline. Filtering pipeline were filtering mutations based on sequencing evidence (VAF, coverage, mutation likelihood TLOD and NLOD, strand bias, allele depth, and multiallelic variants), overlap in problematic regions including regions with low mappability and repetitive sequence, and homopolymer repeats. The resulting set of somatic variant calls included both on- and off-target mutations.

### Somatic variant calling QC

During manual review of variant calls for each sample, we identified 71 cases in which a minority of likely somatic variants were misclassified as germline or noise because of possible tumor contamination in matched normal samples. To rescue these variant calls, we used DeTiN (30), a tool designed to detect and quantify TIN contamination and reclassify variants in which read evidence in the normal is consistent with contamination. We used DeTiN with the default settings of

--mutation_prior = 0.05, --aSCNA_threshold = 0.1, --TiN_prior = 0.5, --resolution = 101, --ascna_probe_number_filter = 200, --ascna_SNP_number_filter = 20, --coverage_threshold = 20, --SSNV_af_threshold = 0.2, and --aSCNA_variance_threshold = 0.025.

Results of DeTiN can be found in Supplementary Table S1.

### Normal sample selection

For several of the patients in our cohort, we had multiple matching normal samples available. For CN variation (CNV) analysis, we pooled all the normal samples available for each patient into one sample and used the pooled sample for our CNV analysis. For somatic mutation calls, for each patient we used DeTiN to identify the normal sample with the least contamination in tumors and then used that matching normal sample to call somatic mutations for all the samples of the patient. A list of normal samples used in somatic mutation calls can be seen in Supplementary Table S2.

### Absolute CN analysis

CN analysis was performed by using whole-exome sequencing (WES) coverage data and variant calls based on the tumor DNA. CNVEX (25, 26) was used to estimate CNVs. Briefly, CNVEX estimates coverage within fixed genomic intervals and variant calls to compute BAFs at variant positions. Coverage values are then normalized for GC bias using LOESS smoothing across targeted regions within the GC range of 0.3 and 0.7 using the span = 0.5. All the GC-normalized coverages and BAFs are then jointly segmented using a custom algorithm based on circular binary segmentation (31). The resulting segmented CN profiles were then subjected to joint inference of tumor purity and ploidy and absolute CN states, implemented in CNVEX, which is most similar to the mathematical formalism of ABSOLUTE (32) and PureCN (33). Because the CN inference problem can have multiple equally likely solutions, further biological insights are necessary to choose the most parsimonious result. The solutions were reviewed by two independent field experts, and the most likely solution was selected.

Using the absolute CN states, we defined absolute baseline CN of a tumor as the weighted median CN of its segments (weighted based on the length of the segment). Next, we defined CNV events as follows:

- Segment gain: any segment with CN higher than the baseline CN.
- Segment amplification: any segment with at least seven copies and CN higher than two times of the baseline CN of the chromosome it is located on.
- Segment deletion: any segment with CN lower than the baseline CN.
- Segment homozygous deletion: any segment with the absolute CN of 0.
- Segment LOH: a segment with hemizygous loss, copy-neutral LOH, or multiple copies of the same allele. Hemizygous deletions and LOH have been combined in our analyses, as we have not observed instances of copy gains following LOH affecting the mutant allele of a gene with the known pathogenic role in prostate cancer.

### WES QC

Total number of reads, percent of duplicated reads, and percent of chimeric reads were calculated for WES and RNA data using the same criteria as Picard (https://github.com/broadinstitute/picard; Supplementary Fig. S1A and S1B). We confirmed that these quality measures did not affect the downstream analysis, including estimation of purity and ploidy derived from CN and germline variant calls (Supplementary Fig. S1C and S1D). Other quality measures such as GC bias (33) and read mappability were factored directly in the CNV and variant calling processes to minimize the effects of data quality on sensitivity of the calls. Tumor purity can negatively affect the accuracy of both CNV detection and inference of purity and ploidy. We have examined the possible influence of tumor purity on the detection of CNV segments and absolute CN analysis (Supplementary Fig. S1E). We noted that in samples with <25% purity, the number of observed CNV segments indicates a loss in sensitivity. However, metrics based on absolute CN inference were not significantly affected (Supplementary Fig. S1F).

### RNA-seq expression quantification

For RNA-seq data analysis, including read trimming, alignment, postprocessing, and quantification, we used the same methods as previously described (26). Briefly, adapter-trimmed paired-end 150 bp short reads were aligned using STAR (34) version 2.4.0j to the GRCh38 reference supplemented with human oncogenic viruses. To improve the sensitivity of detecting fusion calls, synthetic single-end reads were generated using bbduk from the trimmed paired-end reads. These reads were also aligned using STAR with optimized settings. After alignment, capture RNA-seq data were quantified using Kallisto (35). The paired-end and single-end RNA-seq alignments were used subsequently as an input to CODAC, a component of TPO designed to call fusions, perform additional QC, and realign reads using Minimap (36) and GMAP (37).

### Pathogenic alterations

In this article, we refer to an alteration as “pathogenic” if the alteration is happening in one of the recurrently altered genes in mCRPC and causes pathogenic phenotypes such as oncogenesis (e.g., inactivation of *CDK12* or homozygous deletion of *PTEN*), progression (e.g., alterations in *TP53*), or acquired resistance (e.g., amplification or hotspot mutations of *AR*). A comprehensive list of genes with possible pathogenic alterations can be found in Supplementary Table S3, which was previously proposed by Robinson and colleagues (21). It is important to note that for some of the genes proposed by Robinson and colleagues, we did not observe any mutations in our cohort (e.g., *SPOP*). Those genes are excluded from the list of genes in our analysis. Overall, the frequency of somatic alterations is similar to those reported before for mCRPC (Supplementary Table S3).

### Tumor mutational burden calls

Tumor mutational burden (TMB) was defined, as previously proposed (38), as the total number of nonsynonymous somatic mutations (see Somatic variant calling QC) per megabase of targeted regions (Agilent V4 Exome). No additional VAF threshold was used. Mutation types included in the calculation include missense, nonsense, frameshift, and splice-site variants.

### Tumor CN burden (wGII)

wGII (39) is a method used to calculate the CN burden of the genome. To calculate wGII, we first measured the absolute baseline CN of the genome (explained above). Next, we calculated the portion of the genome with a CN other than the absolute baseline CN per each chromosome. Lastly, we took an average of the altered portion of each chromosome to find the genome-level CN burden.

### Tumor purity variability statistical tests

We evaluated the statistical association of tumor purity estimates (see Somatic variant calling QC) separately with patient identity and tumor tissue site using a one-way ANOVA test.

### Detection of recurrent amplifications and deletions

To detect loci with recurrent amplifications and deletions in prostate cancer, we ran GISTIC 2.0 (40) on the CAPSTONE cohort (41) consisting of 325, almost exclusively metastatic, prostate cancer samples. We ran GISTIC 2.0 using the following parameters:

-rx 0 -js 4 -broad 0.98 -cap 1.5 -twoside 1 -genegistic 1 -smallmem 0 -conf 0.99 -armpeel 1 -savegene 1 -saveseg 1 -qvt 0.1.

### Cancer cell fraction calculation

The cancer cell fraction (CCF) of each mutation was calculated similar to the method explained in (42). In short, CCF is a value between 0 and 1, which estimates the proportion of tumor cells harboring a particular somatic mutation. CCFs were calculated in two steps: (i) estimating the multiplicity (*M*) of the mutation (i.e., how many alleles in the tumor harbor the mutation) and (ii) normalizing VAF for *M* and tumor purity. To calculate *M*, the allele count with the maximum likelihood (L) was selected according to the following equation:

$$M_{L}\;=\;Beta\left(Ef,\;ADT,RDT\right),$$

where

$$Ef=\frac{Allele-count_{mut}}{\left(Allele-count_{mut}+Allele-count_{wt}\right)+Allele-count_{normal}}=\frac{P*M}{P*C\;+\;\left(1-P\right)*nC}$$

In the equations above, androgen deprivation therapy (ADT) is the alternative read count in tumor (i.e., number of reads supporting the variant), RDT is the reference read count in tumor (i.e., number of reads supporting the reference at the variant position), *P* is the purity of the sample, *C* is the total absolute CN of the locus harboring the variant, and *nC* is the germline CN of the locus harboring variant 2 for autosomes; in males, it is 1 for chrX and 1 for chrY, whereas in females it is 2 for chrX and 0 for chrY. Using the equation (A), we are able to pick the *M* that maximizes the β likelihood.

$$\hat{m}\;=\;\arg\;max_{M\;\in \;\left\{1,...,C_{i}^{A}\right\}}\;\;Beta\left(\frac{P*M}{P*C\;+\;\left(1-P\right)*nC},\;ADT,RDT\right)$$

After estimating *M*, CCF was calculated as

$$CCF\;=\;\frac{AFT*nC*\left(1-P\right)\;+\;C*P}{\hat{m}*P},$$

where AFT is the alternate allele frequency in the tumor. Any mutation with CCF >0.87 was considered to be clonal. This threshold was used as a marginally relaxed version of thresholds previously used by other publications (43, 44). We relaxed the threshold to better capture the left tail of the normal distribution of CCFs around CCF = 1.

### Mutation classification according to presence and clonality in patients

We classified somatic mutations into five distinct categories. The first category, termed “truncal,” encompasses mutations that are clonal and ubiquitously present across all metastatic tumors in a patient. These mutations are indicative of early oncogenic events, present in the common ancestral clone giving rise to all subsequent metastatic tumors. In patients from whom we have taken a sample from the primary tissue (i.e., prostate), we can conclude that these mutations were present in the primary tissue and they have most likely formed before the metastasis dissemination. In patients without the tumor from the primary tissue, we can only indicate the presence of these mutations in the most recent common ancestor (MRCA).

The second category, “clonal-shared,” includes mutations that, although clonal in at least one site, are also detected in more than one location but are not truncal. These mutations arise after truncal mutations and are not present in the common ancestor, with the associated clone most likely seeding in at least one other metastatic site.

“Subclonal-shared” mutations constitute the third category. These mutations are present in multiple sites but never reach clonal dominance, suggesting a pattern of migration between sites for their associated clones rather than seeding any metastatic site.

The final two categories are “private” mutations, unique to a single site, and “clonal-private” mutations that are clonal within their specific site, likely representing either a seeded clone from another site where the source is not captured because of undersampling or a mutation that originated and clonally expanded within the same site. “Subclonal-private” mutations, in contrast, either developed within their observed site without achieving clonal status or migrated from an unsequenced source site.

### CNV classification according to presence in patients

For the study of CNV heterogeneity, we grouped CNVs into two main groups as such:

- If a region is gained/lost in all of the tumors from a patient, we call that event a truncal gain/loss, as it is a shared event among all the tumors.
- If a region is gained in one or more tumors but is neutral or lost in any of the patients’ tumors, then we call it a heterogeneous gain. Similarly for a loss, if a region is lost in one or more tumors but is neutral or lost in any of the patients’ tumors, then we call it a heterogeneous loss.

### Mutational cluster detection

The emergence of a new tumor clone, marked by a distinct set of mutations, results in similar CCF values for these mutations, as they exist in the same subset of cancer cells. This pattern is detectable through clustering the CCFs for each patient, a process we conducted using the PhylogicNDT (bioRxiv 2019.02.16.508127v2).

Using the calculated CCFs, the PhylogicNDT cluster module was used to cluster mutations based on their CCF values among all the samples from the same patient. The PhylogicNDT cluster module was run using the following setting:

-ni 500 -rb --Pi_k_mu 2 --Pi_k_r 10.

The resulted clusters were iteratively pruned according to the following criteria:

- Each cluster was required to be supported by at least three mutations.
- If there exists a cluster with the mean CCF difference of <10% from another cluster in all samples of a patient, the two clusters are merged together, and a new mean CCF for the merged cluster is calculated using the mean of all the mutations in the new cluster per each sample.

### Mutational cluster verification using whole-genome sequencing

We verified the utility of whole WES for the detection of clusters in patients. We selected all the samples from two of the patients to perform whole-genome sequencing (WGS) and performed mutation calling and inferring clonal structure of tumors.

We selected patients WA_55 (4 sites) and WA_56 (4 sites) that had high purity (Supplementary Table S4) and were representative in both the numbers of clones and mutations for a more generalizable comparison between the two strategies. Using the similar sequencing protocol mentioned above, WGS of the samples yielded an average depth of 60X [95% confidence interval (CI), 56X–63X]. We used the same mutation calling method as WES and compared the inferred clonal structure based on WES and WGS. PyClone-VI was used to infer the clonal structure of WGS samples, as it is widely used for clonal interference of WGS data (14). We observed that the major clones that harbor the majority of mutations detected in WGS samples can be mapped to clones detected using WES. For WA_55, 10 of 20 clones detected in WGS mapped to clones previously detected using WES. Notably, these clones harbor 87% of all the mutations detected in WGS. Similarly, for WA_56, 9 of 21 clones detected in WGS mapped to clones previously detected using WES. Although in this patient the majority of clones were not detected in WES, the ones that were consistent with WES clones harbored 93% of all the mutations detected using WGS. Overall, these data suggest that WES can capture clonal complexity attributable to ∼90% of all the mutations detectable in WGS. Furthermore, these data suggest that the higher number of mutations detected by WGS does not necessarily contribute to detecting more clones as ∼90% of them are attributed to the clones previously identified in WES.

### Categorizing clones according to their position in phylogenetic trees

We sought to categorize clones in each patient based on the location of mutation clusters within the phylogenetic trees into three distinct groups: truncal, branch, and leaf.

“Truncal” clones were defined as any cluster located at the trunk of the tree. Mutations in these clones were present and clonal across all patient sites. Given that mutations in truncal clones exist ubiquitously across all samples and consistently exhibit clonal characteristics, they can be perceived as mutations that are present in the MRCA of all the clones, and in patients with the primary tumor available, we can conclude that they have likely occurred prior to the invasive state of the tumor, which leads to distant tissue metastasis.

“Leaf” clones, by contrast, referred to clusters that were exclusive to one site. Such clones, private to a site, provide insights into the variability and potential adaptability of tumor cells.

Finally, a third group of clones labeled as “branch” comprised all clusters that did not fall within the truncal or leaf classification. These clusters would typically be found in more than one site, which will place them in between or along the branches of the tree, hence the aptly named category. Branch clones can be explained as clones that occurred after the dissemination of clones from their common ancestor but also were involved in the metastasis process and therefore their presence in more than one site.

### Measuring the extent of “illusion of clonality”

To measure the extent of the “illusion of clonality,” we looked at each mutation within a patient separately. For each patient’s mutations, we measured in what proportion of samples from that patient the mutation is clonal ($P_{C}$):

$$P_{C,i}\;=\;\frac{N_{C,i}}{N_{A,i}}$$

In the equation above, $N_{C,i}$ corresponds to the number of samples in which mutation i is present and clonal. $N_{A,i}$ corresponds to the number of samples in which mutation i is present (clonal or subclonal). If a mutation was not present in a sample, we did not include that sample in the denominator of $P_{C,i}$, as an absent mutation would not cause the illusion of clonality. Next, we averaged over all the $P_{C,i}$ values for all the mutations to derive a clonality proportion at the patient level. Depending on the number of samples that are being evaluated for each patient, $P_{C,i}$ values can be different. We repeated the same procedure for combinations of two, three, and four samples from each patient. In the cases of more than one sample, if a mutation is clonal and present in all the samples, it will be called clonal; otherwise, we consider it as subclonal. In the end, we have a clonality rate for each patient per number of evaluated samples.

### Estimating event detection rates

To calculate the detection rates of mutations and CNVs per different number of samples available, we first recorded all the mutations and CNVs that were detected using all samples of each patient. Next, we permuted all the two, three, and four pairings of samples within each patient and evaluated if the patient’s mutations or CNVs are observed in the pairing. Depending on the mutation or CNV of interest, we calculated the proportion of two, three, and four pairings in which the alteration of interest is observed. Lastly, to derive a detection rate for each alteration independent of patients, we averaged over all the detection rates derived for each patient.

### Unobserved mutation extrapolation model

To extrapolate the number of unobserved mutations in each patient in the unsequenced tumors, we used the model explained in (45). Deng and colleagues propose a nonparametric model that can robustly predict the number of unobserved clones for long-range extrapolation based on the number of mutations already observed in the sequenced tumors. The model aims to extrapolate the species accumulation curve in order to compare the diversity of populations based on samples of differing sizes. This class of models has previously been employed in other biological applications such as predicting the unseen variants in the human genome (46).

We used the implementation of this method in function ds.rSAC.bootstrap() in the preseqR package (45) to perform extrapolation using the r = 1; mt = 20 settings. We ran the model on all the patients separately using the available samples for each corresponding patient. From there, we found the predicted number of mutations and clones per each additional number of samples for the patient. The number in the model corresponding to 100 samples was used as the maximum number of mutations observable in each patient.

We validated the model on mutation data by selecting all the patients that had at least four sites available. We used three sites to train the model and then made a prediction of the number of total observable mutations for the case of four samples available. We then compared the predicted value with the actual observed value from the data to validate the model performance.

### Reconstruction of clonal phylogenetic trees

Phylogenetic trees were built using CONIPHER (version 0.1.0; ref. 47). In short, CONIPHER uses the pigeonhole principle and infinite sites hypothesis (48) to reconstruct a phylogenetic tree based on CCF clusters of mutations, i.e., clones. In this analysis, we input filtered synonymous and nonsynonymous mutations to the tool. In the process of reconstructing phylogenetic trees, CONIPHER removes any cluster that is driven by CN alterations, does not fit the pigeonhole principle, or might cause a cycle in the tree. To ensure no driver mutation has been removed, all the removed mutations were manually assessed according to a compiled list of mutated genes of interest in prostate cancer same as in Supplementary Table S3. Five variants were detected to have been removed and were present in the list of genes of interest. Those mutations were merged back to the cluster with the closest mean CCF to the variant CCF. Two patients (WA_24 and WA_25) did not have any shared mutations among all their sites, and hence it was not possible to infer a phylogenetic tree for them because of lack of a truncal cluster. WA_24 was left out of phylogenetic reconstruction because of the lack of shared mutations across all sequenced sites, indicating the possibility of metastasis from a clonally unrelated tumor.

### Migration pattern inference

Migration patterns for each patient were inferred according to their phylogenetic trees by employing the principles previously explained using MACHINA (49). Briefly, we used the average CCF to analyze the presence of clones in different sites. If a clone is present in more than one tissue, there must be a migration between the tissues, but the direction is unclear. Given that a seeding clone initiates a new metastatic lesion, the seeding clone will become dominant (CCF = 1) in the new lesion. We used this principle to infer the directionality of migrations; i.e, a seeding clone should have CCF ≤1 in the source site and CCF ≈1. For any other migrations (e.g., migrations to an existing lesion), we inferred the most parsimonious path of migrations (i.e., path with the minimum number of migrations) between two clones.

### LOH permutation model

We used a permutation test to quantitatively assess the statistical significance of the observed overlap in LOH across the four samples from patient WA_24. This test measures the probability of observing concurrent LOH of genes in four tumors, if the LOHs are happening in random loci of each tumor (i.e., null hypothesis).

The first step of the test involves classifying segments according to their LOH status into two groups of LOH and noLOH. Next, in each tumor, LOH labels were uniformly shuffled among segments to simulate a randomly labeled genome. To make sure that the rate of LOH in the simulated genome is equal or higher than the tumor (to achieve an upper bound of LOH overlaps), we augmented 20% more LOH labels in each tumor and removed any simulation that has less LOH in their genome compared with the tumors. This simulation was repeated a million times, and in each step the number of overlapping genes with LOH in all four simulated samples was measured. The observed number for genes with LOH in all four samples of patient WA_24 was then compared against this simulated distribution, yielding an empirical *P* value.

### Inclusion and ethics statement

This research was conducted with a commitment to inclusivity and ethical standards. Ethical guidelines for patient enrollment were strictly followed, prioritizing integrity, transparency, and respect throughout the research process. We aim to contribute to a scientific community that values and upholds these principles.

## Results

### Multisite sequencing of a rapid autopsy cohort of mCRPC

**Figure 1.**
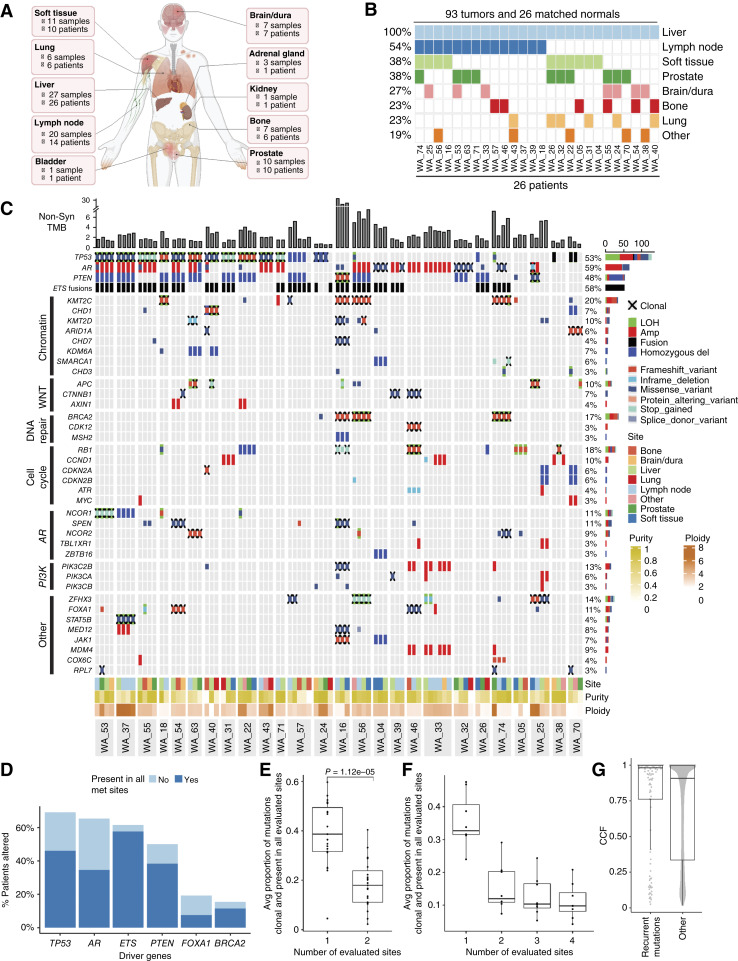
Overview of the cohort and ubiquitous alterations across metastatic tumors in mCRPC. **A,** Overview of the cohort by number of patients and samples obtained from each tissue. **B,** Patients and their corresponding sequenced tissue sites. Some of the patients might have multiple tumors from the same site. The “other” category includes tissues for which fewer than six tumors are obtained. **C,** Alteration landscape of the cohort. Likely driver genes, which are commonly altered in prostate cancer and altered at least once in this cohort, are included. LOH is only shown when it is accompanied by a mutation. **D,** Proportion of patients with alterations in genes recurrently altered in prostate cancer (alterations shown in **C**). Among CNV alterations, only homozygous deletions and amplifications have been considered. **E** and **F,** Proportion of mutations classified as present and clonal in all sites as a function of the number of sites evaluated. **E,** All patients. **F,** Patients with at least four sites sequenced. Significance based on the Wilcoxon rank-sum test. **G,** CCF of mutations in prostate cancer recurrently mutated genes compared with all other detected mutations. Amp, amplification; Avg, average; del, deletion; met, metastasis, Non-Syn, nonsynonymous.

To understand and characterize intrapatient (2) heterogeneity in mCRPC, we performed matched tumor–normal whole-exome DNA sequencing (WES) and RNA-seq (Supplementary Tables S5 and S6) on a rapid autopsy cohort of 26 patients (Fig. 1A; Supplementary Table S7), all with extensively metastatic castrate-resistant disease, for a total of 93 tumors (median: 3 metastatic sites per patient, Fig. 1B; Supplementary Table S2). We profiled the matched tumor and normal samples to an average depth of 590X (95% CI, 553X–627X) and 520X (95% CI, 360X–680X), respectively (Supplementary Table S8). We performed extensive QC (Supplementary Fig. S1A–S1D), focusing on library complexity and sequencing coverage (Supplementary Tables S8 and S9), tumor purity, and TIN contamination (30), and excluded three samples with low tumor content (purity <10%). Utilizing the WES data, we called somatic variants (Supplementary Table S10; Materials and Methods), inferred CNVs (Supplementary Table S11; ref. 33), and estimated tumor purity and ploidy (Supplementary Table S4). The CNV models were independently manually reviewed for goodness of fit, and we further confirmed that purity is not systematically affecting the rate of mutation (including single-nucleotide variants and short insertion and deletion) or CNV calls. In our cohort, overall, a weak correlation (R2 = 0.048; *P* = 0.045) between TMB and purity was observed, which may reflect limited sensitivity of variant calling below 25% tumor purity, which affects six samples. At the patient level, only 2 of 26 patients showed a significantly positive correlation between purity and TMB or wGII (Supplementary Fig. S1E). For CNV, we noted a decrease in the resolution of detected CNVs for 6 (7%) samples with <25% purity (Supplementary Fig. S1F).

By analyzing independent tumor samples across multiple patients, we aimed to investigate the impact of shared cancer biology (e.g., differences in intrinsic immunogenicity of each cancer) on tumor purity and the tumor microenvironment (TME; Materials and Methods). We observed that although tumor purities are highly variable, both within (average IQR = 0.15) and across patients (IQR = 0.33), they are strongly associated with both the patient (*P* = 0.0011; Supplementary Fig. S1G) and tissue site (*P* = 0.0071; Supplementary Fig. S1H), with lung and bone tissues having systematically the lowest purity. Lower purity in lung lesions has been previously attributed to increased immune cell infiltration in TME (50, 51). We investigated the TME of lung tumors and observed a significant increase in the infiltration of neutrophils in lung tumors compared with other sites (*P* = 0.0007; Supplementary Fig. S1I). In agreement with prior studies (21), our cohort revealed a high incidence of nonsynonymous genetic alterations in key genes associated with late-stage mCRPC, notably mutations in *TP53*, *AR*, *PTEN*, and *ETS* fusions (Fig. 1C). *ETS* fusions were identified using RNA-seq (Materials and Methods) in 62% of our cohort, with a notable 58% of patients harboring these fusions across all of their metastatic sites (Fig. 1D). *BRCA2*, an actionable target in mCRPC, was present in 15% of patients, with 75% (3/4) of patients harboring *BRCA2* mutations clonally (i.e., CCF >0.87; Materials and Methods) in all sites and one patient harboring a likely passenger missense mutation. In contrast, *TP53* mutations were identified in 69% of patients, but they were present across all metastatic sites of only 46% of patients (Fig. 1D). A similar pattern was observed for *AR* alterations, in which we found 65% of patients having *AR* alterations, but only 35% of them had the alterations present in all sites. Notably, two patients harbored a clonal hotspot *AR* mutation (Thr878Ala) and *AR* amplification, respectively, only in one of the samples but not in the rest, indicative of clonal sweep or reseeding. However, another patient had a subclonal hotspot mutation (His875Tyr), which was not observed in any of the other sequenced metastatic sites.

### Sequencing a single metastatic site overestimates clonality of mutations

“Illusion of clonality” (14, 52) refers to the phenomenon in which a mutation occurs as clonal if only a single sample is sequenced, but the mutation is either absent or subclonal in other metastatic sites. To examine the clonality of mutations, we quantified the CCF for each mutation (Supplementary Fig. S1J; Materials and Methods), the majority being likely passenger mutations, subsequently classifying them as clonal or subclonal based on their CCF values (Materials and Methods). When a single sample is evaluated, 38% (95% CI, 34%–45%) of mutations occur as clonal; however, if pairs of samples are jointly evaluated, the proportion of mutations that are present and clonal in both sites drops to 18% (95% CI, 14%–23%; *P* = 1.12e–5; Fig. 1E). To extrapolate this trend to widespread metastatic disease, we analyzed patients with at least four tumor sites sequenced. The rate of clonal mutations that are present in all evaluated sites decreased from 36% (95% CI, 26%–45%) with one evaluated sample to 15% (95% CI, 6%–18%) with two and 11% (95% CI, 3%–12%) with four, showing that sampling more than two sites provides little benefit in identifying clonal mutations (Fig. 1F).

Next, we investigated the illusion of clonality for likely pathogenic mutations in genes recurrently altered in mCRPC among patients with four samples sequenced. For *TP53*, the proportion of ostensibly clonal mutations detected when a single site is sequenced was 58%. However, for 43% of patients, all the mutations present across patient sites were clonal (Supplementary Fig. S1K). More strikingly, although *AR* mutations were identified as clonal in 50% of patients with *AR* mutations present using a single sample, only 20% of patients harbored clonal *AR* mutations in all sites. We observed that likely pathogenic mutations had the highest average CCF, with the majority being clonal (CCF >0.85; Fig. 1G). *CDK12*, *PTEN*, and *TP53* hotspot and truncating mutations were also among the mutations with highest CCF, whereas mutations in *AR*, *APC*, and *FOXA1* displayed a bimodal distribution of CCFs, suggesting their both clonal and subclonal prevalence within tumors (Supplementary Fig. S1L). These patterns reinforce the observation that *AR* and Wnt pathway mutations are more common after dissemination as a heterogeneous resistance response to treatment (7), whereas *TP53* mutations may arise in the most recent common ancestor (MRCA) clone and likely preceding metastasis (53).

### Most mutations in mCRPC are subclonal and restricted to a single site

**Figure 2.**
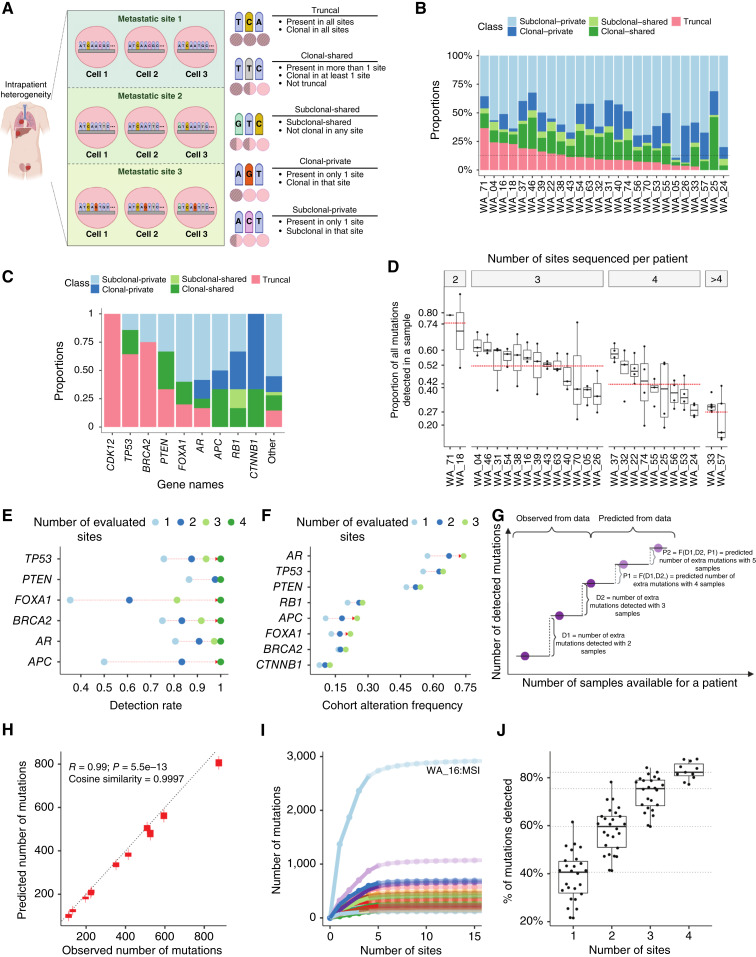
Mutational heterogeneity in mCRPC and its impact on detecting alterations using a single sample per patient. **A,** Schematic representation of mutation classification used in this study. Left, three metastatic sites from a single patient with tumor cells in each site. Mutations present in each cell are indicated by colors other than blue. Right, mutations alongside their classifications. Each pink circle on the right corresponds to one of the metastatic sites. A fully hatched circle indicates a mutation that is present in all the cells within the site (i.e., clonal), a partially hatched circle signifies a mutation that is present in some but not all of the cells within the site (i.e., subclonal), and a clear circle shows the absence of any mutation. **B** and **C,** Proportion of mutations in each class by each patient (**B**) and prostate cancer recurrently mutated gene (**C**). Other represents all remaining observed mutations. **D,** Fraction of mutations identified in each individual sample relative to all mutations for that patient in all sites, stratified by the number of sites profiled for a patient. Red dashed lines represent the average proportion of mutations detected. **E,** Detection rate of alterations for each gene. In this analysis, only patients with at least four sequenced tumors are assessed. The detection rate when evaluating four sites is set as a baseline of one for each gene, and the relative detection rates when fewer than four sites are evaluated. **F,** Impact of profiling additional tumors on cohort level observed prevalence of alteration in prostate cancer recurrently mutated genes. Only patients with three sequenced tumors were assessed. **G,** Schematic of the “species accumulation model” extrapolating the number of additional mutations that will be detected when another site is sequenced. The model predicts one step ahead, using all previously observed (solid) and predicted (semitransparent) new mutations. **H,** Validation of predictions from the model in **G**. The *x*-axis shows the observed number of mutations in four samples. Each *y*-axis box displays the IQR of the corresponding prediction based on the data from the first three sites. **I,** Results for the model in **G**. Observed (solid) and predicted (semitransparent) numbers of new mutations per additional site profiled. Each curve represents an individual patient. MSI, microsatellite instability. **J,** Proportions of mutations detected as increasing numbers of sites are evaluated. Each dot represents the percentage of mutations detected in a group of samples evaluated. The maximal number of mutations per patient corresponds to the asymptote from **I**.

The clonality of a mutation provides information about the fitness and expansion of cells harboring that mutation, whereas the presence of a mutation across different sites indicates whether the mutation occurred before or after the dissemination of clones from their common ancestor. Accordingly, we categorized mutations into five groups (subclonal-private, subclonal-shared, clonal-private, clonal-shared, and truncal) based on their clonal status and distribution across metastatic sites (Fig. 2A; Supplementary Fig. S2A; Materials and Methods). We observed that subclonal-private mutations were the most common in our cohort, accounting for 55% of all the mutations, whereas truncal mutations accounted for only 13% (Supplementary Fig. S2B). This demonstrates that the majority of mutations in mCRPC occur after the dissemination of tumors from their MRCA. Although many of those mutations are associated with rapidly expanding clones (clonal sweeps), likely the majority are passengers. Mutations present in some, but not all, sites i.e., clonal-shared and subclonal-shared, were observed in patients at varying proportions of 15% and 3%, respectively (Fig. 2B), which is indicative of complex phylogenetic and dissemination patterns. Clonal mutations observed in only one site, i.e., clonal-private mutations, which can be indicative of clonal sweeps, accounted for 14% of all mutations. Finally, we noted the near-absence of truncal mutations in some patients (Fig. 2B), e.g., WA_24, which we examine in detail later.

We extended mutational classification to likely pathogenic mutations in genes commonly altered in mCRPC (21). We observed that *CDK12* mutations were all truncal (Fig. 2C). This was expected, as *CDK12* mutations have been reported to be early drivers of prostate cancer (54). Moreover, we observed that mutations in *TP53* were truncal in more than half of the patients (Fig. 2C), indicating that despite the tendency of pathogenic *TP53* mutations to emerge later in the tumor’s evolution (21), a significant proportion occurred prior to tumor clonal dissemination from MRCA. Conversely, mutations in the *AR* gene, which similarly tend to arise later in the tumor’s lifespan, were truncal in fewer than 20% of cases (Fig. 2C). This suggests that *AR* mutations predominantly occur following tumor spread, consistent with previous reports (17).

### Single-site sequencing underestimates patient- and cohort-level mutation rates

Given the common clinical practice of characterizing metastatic tumors using a single biopsy, we evaluated what proportion of all mutations (including known pathogenic and likely passenger) present across multiple metastatic sites would be detected if only one sample is sequenced (i.e., per-sample detection rate; Materials and Methods; Fig. 2D). As more sites are sequenced, the detection rate decreases from 74% (95% CI, 94%–54%) for one additional site to 27% (95% CI, 21%–33%) for four or more. We noted significant variability in those estimates for both individual sites and across patients. Therefore, we explored mutational concordance of tumor pairs, i.e., the proportion of mutations shared between any pair of tumors for each patient. We observed concordance from as high as 60% to as low as 2% (Supplementary Fig. S2C). These trends underscore that for most patients with mCRPC, analyzing a single metastatic tumor site would miss the majority of mutations.

Next, we evaluated how profiling one compared with multiple sites per patient affects the chance of detecting an alteration (i.e., per-patient detection rate; Materials and Methods). For likely pathogenic genes in mCRPC, we observed an increased detection rate as the number of sampled sites increased (Fig. 2E). The per-patient detection rate increased the most for *FOXA1* and least for early alterations such as *PTEN* (Fig. 2E). Notably, *TP53*, *BRCA2*, and *AR* alterations remained highly detectable even from a single sample, whereas those in *FOXA1*, *RB1*, and *APC* benefited from sequencing multiple sites. Similarly, sampling a single site results in an underestimation of cohort-wide alteration frequencies, most notably for *APC* and *AR* (Fig. 2F).

Next, we employed a mathematical framework to model the expected yield of mutations with increasing numbers of sequenced samples (Fig. 2G; Materials and Methods). The model draws on the similarity between calling mutations in multiple tumors and sampling species at distinct ecologic sites (55). In this application, the model extrapolates the (unknown) lower bound of the total number of mutations per patient (Materials and Methods), which enables the calculation of the proportion of detected mutations as a function of the number of sequenced sites (Fig. 2G). We evaluated the model’s performance in predicting the number of new mutations in an unseen sample and noted a high correlation between the predicted and actual count (R = 0.99; *P* = 2.5e–13) with a cosine-similarity score of 0.999 (Fig. 2H). By extrapolating the model to a higher number of samples, we reach an asymptote, which can be used as a proxy for the lower bound of the total number of mutations harbored by each patient (Fig. 2I). It is evident that this constitutes a lower bound, as each site will harbor a number of private mutations (average, 71 mutations; 95% CI, 52–91; Supplementary Fig. S2D). We evaluated the proportion of detected mutations (relative to the asymptote) as a function of the number of sequenced sites and found that sequencing a single sample on average uncovers only 40% (95% CI, 35%–43%) of a cancer’s mutations (Fig. 2J; Supplementary Fig. S2E), corroborating the previous results that on average, less than half of a patient’s mutations are captured using a single sample (Fig. 2D). However, the yield increased to 82.2% (95% CI, 80%–85%) with the inclusion of four samples.

### Most CNVs occur after metastasis, but pathogenic alterations are largely truncal

**Figure 3.**
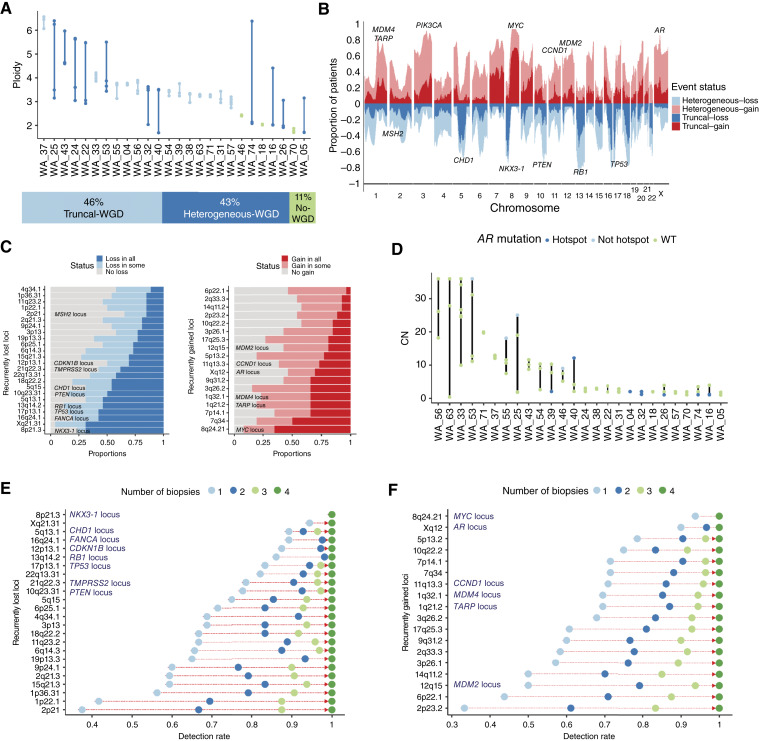
Heterogeneity of recurrent prostate cancer CN alterations across metastatic sites. **A,** Top, heterogeneity in tumor ploidy across metastatic sites. Bottom, proportion of patients with truncal, heterogeneous, or no observed WGD events. **B,** Cohort-wide frequency of CN gains (red) and losses (blue). **C,** Proportion of patients with losses (left) and gains (right) on recurrently deleted and amplified regions in mCRPC. Shading indicates truncal (dark) and heterogeneous observations (per-patient). **D,** Heterogeneity of AR CN and mutation status across metastatic sites. Blue, presence of *AR* mutations. Absolute copy estimates are capped at 35. WT, wild-type. **E,** Increase in the detection rate of CN losses as a function of the number of sites sampled. Select loci recurrently altered in prostate cancer are highlighted. **F,** Increase in the detection rate of CN losses as a function of the number of sites sampled. Select loci recurrently altered in prostate cancer are highlighted.

We extended our analysis of intersite mutational heterogeneity to CN alterations. First, we focused on whole-genome duplication (WGD), which is present in more than 50% of metastatic cancers (56) and typically occurs late in disease progression. We noted that 89% of patients had WGD in at least one of the sites (Fig. 3A; Supplementary Fig. S3A). However, for 43% of patients, the baseline ploidy differed between metastatic sites, indicative of WGD occurrence after metastatic dissemination. Conversely, WGD was identical (i.e., likely truncal, Materials and Methods) in 46% of the patients. WGD is associated with an acceleration of chromosomal instability (CIN; ref. 56), and hence we examined the intersite variability of quantitative CIN measures (Materials and Methods). Despite considerable differences in baseline ploidy, measures of CIN [i.e., wGII (39), %LOH, and large-scale state transition (LST; ref. 57)] were consistent across metastatic sites (Supplementary Fig. S3B and S3C). Strikingly, the same holds for TMB, which is similar across metastatic sites despite the significant mutational heterogeneity.

Next, we explored the heterogeneity of CN alterations, including both genome-wide changes and alterations in genes that are recurrently deleted or amplified in prostate cancer (21). Focusing on the truncal status, we categorized CNVs as truncal if gains or losses were present in all samples from a patient and heterogeneous otherwise (Materials and Methods). In genome-wide changes, only 37% of gains and 32% of losses were truncal (Fig. 3B). However, in regions with recurrent CN alterations (i.e., specific genomic regions that are consistently amplified or deleted across multiple cancer samples; Materials and Methods), we noted a significantly higher degree of truncal gains and losses. Specifically, recurrent deletions, including genes such as *NKX3-1* and *TP53*, were truncal 58% of the time compared with 36% for all other genes (*P* < 1e–16; Supplementary Fig. S3D). Similarly, recurrent gains, including genes such as *MYC* and *CCND1*, exhibited truncal gains 34% of the time compared with 30% for other genes (*P* = 0.01). The *NKX3-1* locus had the highest frequency of truncal losses, whereas the *MYC* locus showed the highest proportion of truncal gains (71%). *RB1* and *TP53* losses were also commonly truncal (61% and 68%, respectively; Fig. 3C). Moreover, whereas *PTEN* loss of one or more copies was truncal in 59% of patients, biallelic deletions were truncal in 82% of *PTEN*-deficient patients (Fig. 1C and D). Given the role of PTEN homozygous deletion in aggressiveness of prostate cancer, these results suggest that pathogenic *PTEN* alterations happen more frequently before metastasis.

Given the importance of *AR* alterations in ADT and androgen receptor signaling inhibitor resistance, and their late emergence, often after dissemination (7), we sought to assess their heterogeneity in the context of lethal mCRPC. We observed pronounced *AR* mutational and CN heterogeneity (Fig. 3D; Supplementary Fig. S3E). Amplifications in the AR locus were truncal in 61% of the patients harboring *AR* amplifications, albeit with significant differences in the amplification level (17). Notably, we found that samples with elevated *AR* gene CNs generally harbored fewer *AR* mutations, and interestingly, when present, these mutations were not in recurrent hotspots (Fig. 3D). Conversely, *AR* hotspot mutations occurred predominantly in samples with lower *AR* CNs, and only one of eight hotspot mutations co-occurred with *AR* amplification (Fig. 3D). Patient WA_39 illustrates the divergent pathways to castration resistance, with two of three tumor sites harboring *AR* amplification but a third site manifesting a hotspot mutation (Supplementary Fig. S3E). The expression of AR target genes, such *KLK3*, remained high (average: 1,190.4 FPKM; 95% CI, 969.2–1411.6) in all of the samples. Finally, mirroring previous analyses of mutations (Fig. 2D and E), we assessed the impact of tissue sampling on detecting likely pathogenic CNVs. Within recurrently altered loci, we found that alterations were detectable from a single sample for the majority of patients, e.g., deletions of *RB1* (86%), *TP53* (83%), and *PTEN* (78%) and amplifications of *MYC* (94%) and *AR* (90%). However, additional samples yielded a further average improvement of detections by 9% and 11%, respectively, for deletions and gains (Fig. 3E and F). Although alterations of *AR* are highly detectable from a single sample, these alterations are often distinct and likely emerge independently, as previously reported (7, 17).

### The majority of tumor clones are detected in only a single site

**Figure 4.**
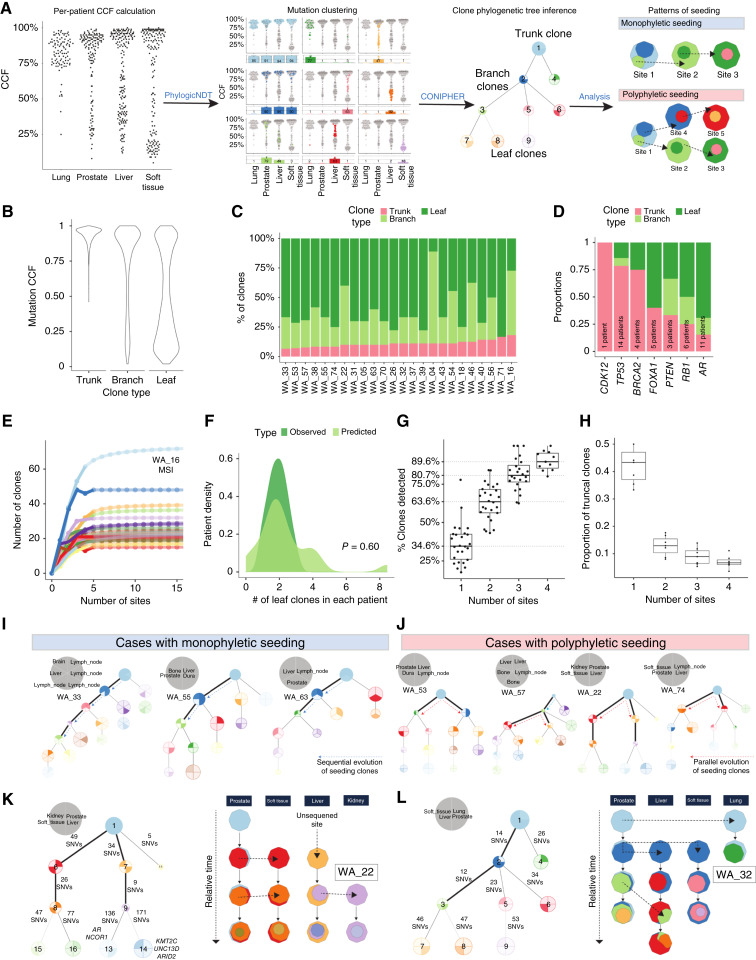
Patterns of clonal evolution and metastatic dissemination in mCRPC. **A,** Analytic workflow for clonal reconstruction, phylogenetic inference, and analysis of metastatic seeding patterns. Each point represents a mutation. **B,** Distribution of CCFs (density) within clone types stratified by position in the phylogenetic tree (trunk, branch, and leaf). **C,** Proportion of clone types by patient. This plot excludes the clones not placed in the trees. **D,** Proportion of clone types by recurrently mutated genes. This plot excludes the clones not placed in the trees. **E,** Total number of detected clones as a function of the number of sites profiled. Each colored curve corresponds to an individual patient. MSI, microsatellite instability. **F,** Number of patients density (*y*-axis) compared with the average number of leaf clones they harbor. Light green, predicted number; dark green, actual number of leaf clones. **G,** Proportion of clones detected as increasing numbers of sites are evaluated. The maximal number of clones per-patient corresponds to the asymptote from **E**. **H,** Proportion of clones detected as truncal (*y*-axis) per different number of evaluated sites (*x*-axis). **I** and **J,** Classification of clonal phylogenetic trees based on ancestral relationships between seeding clones. Bolded edges indicate that the descendant clone seeded a new site. Arrows, parallel seeding (bifurcation). **K** and **L,** Juxtaposition of clonal phylogenetic trees (left) and clonal migration diagrams (right) for two index cases: WA_22 (**K**) and WA_32 (**L**). Clone colors are matched between trees and diagrams. SNV, single-nucleotide variants.

To establish clonal phylogenies and tissue migration patterns, we first identified clones by clustering variants according to their CCF. First, we performed additional WGS of all metastatic sites from two patients to assess the parity of WES and WGS in characterizing the patients’ metastatic disease (Materials and Methods). We observed that in patient WA_55, 10 of 20 clusters detected in WGS map the detected clusters in WES data. Notably, these 10 clones harbored 87% of mutations detected in WGS. Similarly, 9 of 21 detected clusters in WGS were detected in WES, accounting for 93% of WGS mutations. These observations support the utility of WES in capturing the majority of tumor’s clonal complexity (Supplementary Fig. S4A and S4B). Next, using the identified clones, we inferred phylogenetic trees (Materials and Methods) for each patient (Fig. 4A). We successfully inferred phylogenetic trees for 24 of the 26 patients (Supplementary Table S12). We examined the association of metastatic site, purity, TMB, and wGII on clonal heterogeneity (Supplementary Fig. S4C). We noted a significantly lower number of clones in tumors sampled from the lung (*P* = 0.02) and prostate (*P* = 0.04) compared with other tissues (Supplementary Fig. S4D), but no significant differences or notable trends were observed for other tissues. Tumors in the lung and prostate are among the lowest purity in our cohort (Supplementary Fig. S1H). Although we observed fewer clones in these tissues, this finding needs to be further investigated to determine whether it is due to lower detection sensitivity or biological characteristics of these tumor types. This highlights the contribution of ongoing mutagenesis to tumor evolution. These data also suggest that metastatic dissemination to certain tissues may lead to enhanced fitness of dominant tumor clones (i.e., lung metastases), whereas other tissues may promote continued tumor evolution.

Next, we classified clones into three categories based on their position on the phylogenetic tree: trunk, branch, and leaf (Materials and Methods). Mutations from truncal clones are present and clonal across all metastatic sites and have the highest CCF (average CCF = 0.94; Fig. 4B). Branch clones migrate between tissues, being typically clonal at the destination site and either clonal or subclonal at the source sites (average CCF = 0.73; Fig. 4B). Leaf clones emerge after dissemination from the MRCA, are restricted to a single site, and primarily harbor subclonal mutations (average CCF = 0.48; Fig. 4B). Across all patients, the majority of clones were leaf (mean = 61%; 95% CI, 68.1%–53.9%), followed by branch (mean = 29%; 95% CI, 22.2%–35.8%) and trunk (mean = 10%; 95% CI, 11.1%–8.9%; Fig. 4C). The relatively small proportion of trunk clones demonstrates that most clones in patients with mCRPC evolved after dissemination from the MRCA. Additionally, the high proportion of leaf clones in each site indicates that patients have many unique clones per site, leading to considerable heterogeneity across metastatic sites within the same patient.

### Position of driver mutations within the phylogenetic tree is highly variable

Next, we focused on clones harboring pathogenic mutations and their position within the phylogenetic trees. Notably, for more than 75% of the patients, *TP53* mutations occurred in the truncal clone (Fig. 4D). This finding underscores that the majority of these mutations occur prior to dissemination of clones from their MRCA, despite their appearance late in prostate cancer progression. Conversely, mutations in *AR* and *RB1* were truncal in 15% and 25% of cases, respectively, suggesting that these mutations emerge with therapeutic pressure. *AR* mutations, in particular, were predominantly on the leaves of the phylogenetic tree (Fig. 4D), indicating that they were acquired independently in each site. *FOXA1* mutations exhibited a notable pattern, mirroring our previous data in which *FOXA1* mutations showed a bimodal CCF distribution (Fig. 1F). Here, *FOXA1* alterations were either positioned on the trunk or on the leaves of the phylogenetic tree (Fig. 4D). Among the five patients in our cohort with *FOXA1* mutations, three possessed class 1 *FOXA1* mutations (58), with one situated on the tree’s trunk and two on its leaves. Similarly, one class 2 *FOXA1* mutation was located on the leaf and another one on the trunk of their respective phylogenetic tree.

### Single-site sequencing significantly underestimates clonal heterogeneity

We investigate the effect of multisite sampling on the detection of clonal heterogeneity, specifically assessing how many additional unique clones will be detected as more metastatic sites are sampled (Materials and Methods). Utilizing the same mathematical model as for mutations (Fig. 2G), we predicted the total number of clones present in a patient’s tumor ecosystem (Fig. 4E). To assess the accuracy of this prediction, we compared the predicted and observed numbers of additional clones per site, i.e., leaf clones that are only detectable if that site is sequenced. On average, 1 to 2 clones are present in only a single site, with good agreement between the observation and prediction (Fig. 4F). Application of the model revealed that when one sample per patient is sequenced, only 34.6% (95% CI, 31%–42%) of all the tumor clones will be detected (Fig. 4G). This number reaches 89.6% (95% CI, 77%–86%) when four samples are profiled. The determination of whether a clone is truncal is heavily influenced by sampling from multiple and diverse anatomic sites. Similar to the pattern previously described for mutations (Fig. 1F), we observed that based on the analysis of a single site, 40% (95% CI, 36%–44%) of clones would be identified as truncal compared with only 13% (95% CI, 12%–14%) and 6% (95% CI, 4%–8%) when two or more sites are examined, respectively (Fig. 4H). This highlights that dominant clones in one site are often subclonal (or absent) in others.

### Patterns of polyclonal and polyphyletic metastatic seeding

Using the phylogenetic trees, we deduced the evolutionary paths of metastatic seeding, specifically the child–parent relationships of clones seeding or migrating between sites (Materials and Methods; ref. 59). Accordingly, we categorized the phylogenetic trees into three groups. The first group comprised patients in which seeding clones had a child–parent relationship (i.e., monophyletic seeding; Fig. 4I; Supplementary Fig. S4C; ref. 59). Monophyletic seeding was observed for the majority (71%, 17/24) of patients, indicating that the capacity for metastasis had evolved once (Fig. 4A, top right). The second group, accounting for 17% (4/24) patients, had seeding clones located on parallel branches of the tree (Fig. 4J). This pattern of polyphyletic seeding (Fig. 4A, bottom right), initially described in mCRPC by Gundem and colleagues (7), results in high genetic dissimilarity across metastatic sites; e.g., some polyphyletic clones may be more similar to clones in the primary tumor rather than clones in other metastatic sites. In the final group (Supplementary Fig. S4E) of three patients, a single clone seeded all the metastases. However, it is important to note that in this group, patients had fewer sites sequenced compared with the average of our cohort (two patients with two sites and one patient with three sites sequenced). This suggests the possibility that polyclonal seeding is ubiquitous as cases with monoclonal seeding had relatively few sites sequenced.

Additionally, in two patients (WA_22 and WA_32), we observed a pattern of recurrent seeding and migration from the prostate tumor tissue to other metastatic sites. In patient WA_22, we observed initial seeding from the prostate to a soft-tissue lesion (Fig. 4K, left). The data are consistent with a parsimonious model wherein the seeding clone continued to accrue an additional 26 mutations in the prostate evolving into a distinct clone. This clone migrated to the soft-tissue site a second time (Fig. 4K, right) and underwent further parallel evolution in both the prostate and soft tissue, acquiring 77 and 47 distinct mutations, respectively. A similar pattern was observed in patient WA_32 (Fig. 4L, left), in whom clones in the prostate repeatedly migrated to the liver. Following the initial metastasis to the liver, the liver-resident clone acquired 34 new mutations, whereas the progenitor clone in the prostate acquired 12 new mutations, forming a new clone. The newly evolved prostate clone migrated again to the liver, where it acquired 47 additional mutations, resulting in 3 clones (2 in the liver and 1 in the prostate), which share a common ancestor but evolved separately (Fig. 4L, right). Among the nine patients with available prostate tumor samples, recurrent seeding of metastatic sites from the prostate was observed in 2 of 9 patients (Fig. 4I and J; Supplementary Fig. S4C), which suggests an active role for prostate tumors in seeding of metastatic sites over the course of the disease.

### Early metastatic dissemination in a case of neuroendocrine mCRPC

**Figure 5.**
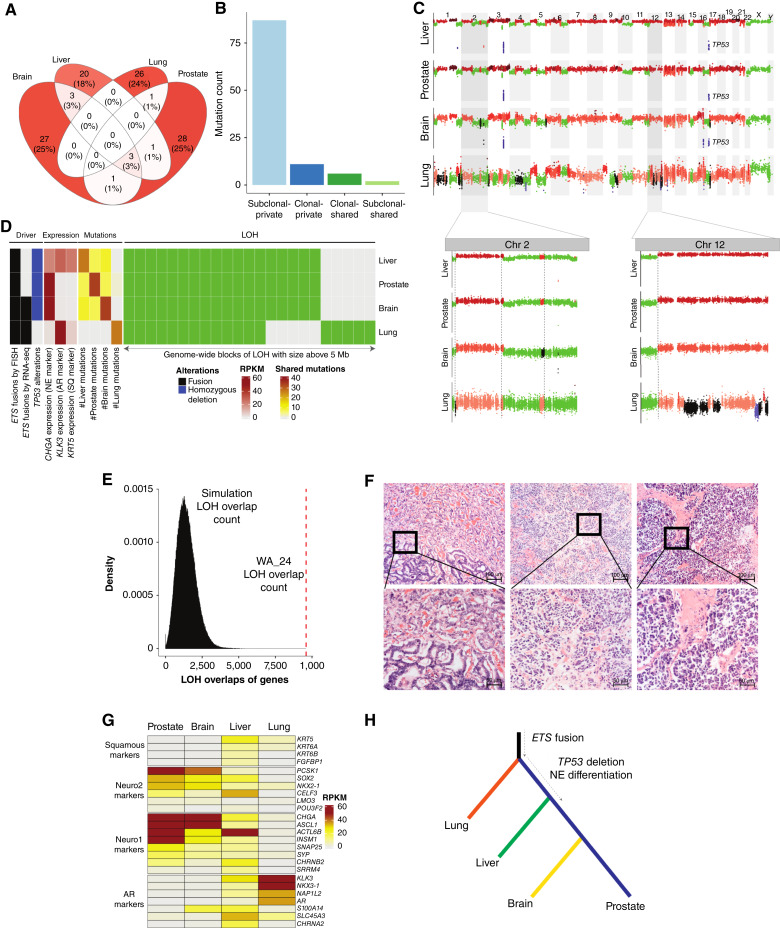
Early metastatic dissemination in a case of mCRPC with heterogeneous NE differentiation. **A,** Overlap of mutations detected in each sequenced site of WA_24. Red color gradient indicates the fraction of all mutations in each intersection. **B,** Tally of mutations classified according to clonality and distribution (see Fig. 2A). **C,** Genome-wide CN profiles of normalized log_2_(tumor/normal) coverage ratios across samples from WA_24 (black, diploid; red, gain; blue, loss; and green, LOH). Two representative regions were selected to illustrate shared LOH segments. **D,** Integrated summary of genetic drivers, gene expression, shared mutations, and notable LOH regions in WA_24 samples. SQ, squamous. **E,** Results of permutation testing comparing the observed number of genes within regions of shared LOH in WA_24 (red dashed lines) with a distribution based on the null model (histogram). **F,** Morphologic heterogeneity and spectrum in WA_24 at different sites with available formalin-fixed, paraffin-embedded slides. Left, mixed classical acinar and organoid pattern in the lung. Middle, small cell NE carcinoma–like pattern in the liver. Right, small cell NE carcinoma–like pattern in the prostate. **G,** Expression of marker genes for molecular subtype mCRPC as defined by Labrecque and colleagues (62). **H,** Parsimonious evolutionary model reflecting the similarity of genetic, phenotypic, and histologic features across tumor sites for patient WA_24.

We identified an index case, WA_24, in which no mutations were shared across all sites (Fig. 5A), raising questions about their phylogenetic relationships. Among all nonsynonymous mutations, 92% (101/110) were subclonal and present in only a single site (i.e., subclonal-private, Fig. 5B), implying their emergence after seeding. The liver, brain, and prostate sites had mutations in common, but the lung site was mutationally distinct. Consistently, the comparison of CNV profiles (Materials and Methods) also revealed higher similarity between the liver, brain, and prostate sites compared with the lung (Fig. 5C). Unlike mutations, CN events can revert to the neutral state or diverge from the common ancestor, rendering them less reliable for inferring tumor phylogenies (60). Conversely, LOH results in irreversible genetic loss that can be used to establish phylogenetic relationships (60). We noted an extensive overlap of regions with LOH across the four tumors, including LOH of the p-arm of chromosome 12 (Fig. 5C). Although the lung lesion displayed a somewhat divergent LOH pattern, the overlap of LOH regions was significant (e.g., 71% LOH overlap between the lung and other sites, Fig. 5D; Supplementary Table S13). To determine whether this could have occurred by chance, we employed a permutation test (Materials and Methods; ref. 61). The observed overlap significantly exceeded all random draws (*P* < 1e–6; Fig. 5E), rejecting the null of coincidence.

Next, to better understand the oncogenesis of this tumor, we sought to identify genetic drivers present in all sites. As no mutations were truncal, we examined the RNA-seq of gene fusions (Materials and Methods) and identified an identical *TMPRSS2*–*ERG* alteration in only two of four samples. However, FISH experiments (19) indicated the presence of this fusion across all sites (Fig. 5D). By investigating this discrepancy, we found that two of four samples in this patient had strong expression of neuroendocrine (NE) markers. Two of these three samples had minimal expression of the AR signaling marker KLK3, most consistent with NE prostate cancer (NEPC; ref. 62). Loss of AR signaling explains the lack of AR-dependent *TMPRSS2–ERG* expression (63). However, one of the three samples retained KLK3 expression—a pattern most consistent with amphicrine prostate cancer that harbors a hybrid cell state that is AR+ NE+ (Fig. 5F and G; ref. 62). This observation explained the lack of *TMPRSS2–ERG* detection in RNA-seq, as NEPC tumors downregulate *TMPRSS2* expression (63). Hence, *ETS* fusions might not always be detectable by RNA-seq. Interestingly, the most genetically distinct lung site from this patient also showed the most strongly *AR*-positive gene expression pattern (Fig. 5F and G). The three *AR* expression– and *AR* signaling–negative sites also harbored a TP53 homozygous deletion, an event often associated with progression to NEPC disease.

Finally, to infer phylogenetic relationships between the tumors, we integrated the irreversible genetic alterations (mutations, LOH events, *ERG* fusion, and *TP53* deletion) alongside the assessment of histologic markers (Fig. 5D and H). The integrative phylogenetic model (Fig. 5H) suggests that the oncogenic process commenced with a cascade of structural alterations including the driver *TMPRSS2–ERG* fusion alongside multiple chromosomal gains and losses. The truncal *AR*-positive clone disseminated immediately to the lung prior to accumulating additional exonic mutations. Within the prostate, the truncal clone acquired a *TP53* homozygous deletion, alongside additional LOH events and three passenger mutations, and metastasized to the liver exhibiting an amphicrine phenotype and to the brain as NEPC (Fig. 5G). This model highlights that in rare instances of mCRPC evolution, acquisition of metastatic potential and lineage plasticity can occur very rapidly.

## Discussion

The genetic heterogeneity of primary prostate cancer has been extensively characterized, with studies reporting multifocality and variable mutation patterns between different tumor foci (4, 10, 11, 64). Other studies have utilized cohorts of primary and local metastatic hormone-sensitive prostate cancer to shed light on mechanisms and drivers of metastasis (65). However, the heterogeneity of mCRPC remains relatively poorly understood. This is important because many providers begin to profile prostate tumors at the time of castration-resistant prostate cancer in order to identify targeted therapy options. Previous studies have uncovered seeding between metastatic sites and clonal evolution patterns (7), dissected the complexity of alterations within the AR locus (17), and shed light on mechanisms and directions of metastasis in mCRPC (15). However, these studies were limited by shallow sequencing depth (≤100X; refs. 7, 17), small cohort sizes (≤10 patients; refs. 7, 15, 17), and focused scope (17).

To address these limitations, we sequenced a set of 93 tumors captured from 26 patients with mCRPC in an effort to characterize the extent of genetic divergence among patient’s metastatic tumors across mutational, transcriptional, and histologic domains. High-depth sequencing facilitated the identification of CN alterations and subclonal mutations, crucial for identifying minor but genetically distinct cell populations. Although we expected high degrees of genetic heterogeneity in mCRPC, the mathematical modeling and phylogenetic reconstruction allowed us to quantify the extent of intrapatient tumor heterogeneity in mCRPC and to characterize each patient’s disease as a clonal ecosystem and evolutionary process, respectively.

Fundamentally, we identified striking levels of genetic heterogeneity for all of the patients. This heterogeneity is driven by both mutations and CN alterations and is manifested at the level of individual mutations and tumor clones. For example, we found that on average, 69% of mutations and 61% of clones in each patient are present in only one site. We also demonstrate that profiling mutations based on a single sample is prone to erroneously classifying mutations as clonal. This phenomenon, known as “illusion of clonality,” is both biologically and clinically (14, 66) significant as it is caused by metastasizing or rapidly expanding clones and is stipulated to be a mechanism for targeted therapy resistance (8). Illustratively, on average only 13% of mutations are truncal, whereas on average 32% of mutations identified as truncal based on a single sample are truncal in all the other sites. Identifying truncal clones and their mutations is crucial for guiding treatment as these mutations are the most suitable targets for durable response in patients. These observations highlight that by evaluating only one sample per patient, we are significantly underpowered to characterize the tumor’s clonal architecture. Similar, albeit not as extreme, trends have been reported in other cancers including non–small cell lung cancer (59) and esophageal squamous cell carcinoma (67).

Underpinning this profound intrapatient heterogeneity are characteristic patterns of clonal evolution. We estimate that the typical mCRPC tumor ecosystem consists of an average 7 (95% CI, 6–8) clones per metastatic site and 13.5 (95% CI, 11–16) clones in total. We note a significant correlation (*R* = 0.78) between TMB and clonal heterogeneity, suggesting that persistent mutagenesis is driving the emergence of new clones. Seeding of metastases is typically polyclonal and often polyphyletic (17%), which manifests in diminished genetic similarity of metastatic lesions and signifies independent acquisition of metastatic potentials (59). Although the population of truncal clones is low in all the samples (except WA_25 in which an independent primary cannot be ruled out), the presence of these clones in all the samples indicates that metastatic clones share a common ancestor and arise from the same lineage in the primary tissue, even though there have been studies indicating the multifocality of the primary prostate cancer (65, 68).

To clarify the implications of these results for mCRPC genetic testing, we examined the heterogeneity of driver alterations and the impact of single-site testing on diagnostic yields. Overall, we found that alterations that initiate prostate cancer or significantly contribute to cancer aggressiveness were largely truncal and benefited least from sampling multiple sites. As previously described (7, 17), we found alterations of *AR* to often be distinct across sites, but somewhat paradoxically the presence of an *AR* alteration in one site was strongly correlated with the existence of equivalent alterations at other sites, indicative of convergent evolution. Conversely, detection rates of alterations in *RB1*, *FOXA1*, and the Wnt pathway tended to improve with multisite sequencing. Both *RB1* and Wnt have been reported to contribute to mCRPC aggressiveness (69). Likewise, facets of aneuploidy, including genome doubling and CN alterations, were also highly heterogeneous, indicative of ongoing CIN. For example, for 43% of patients, baseline ploidy was variable across sites, suggesting that WGD had occurred after metastasis. Tumor purity has been reported to be associated with both intrinsic tumor biology (e.g., specific genomic alterations affecting the TME) and technical challenges of tissue sampling and procurement (50). We found that tumor purity is not merely a technical factor but is biologically informative. Its impact on genomic readouts, such as variant allele frequencies or gene expression profiles, requires therefore especially careful modeling.

Finally, we expanded upon previous observations of multiclonal, metastatic-to-metastatic seeding in mCRPC (7) and identified biologically and clinically relevant examples of tumor phylogenies and tissue migration patterns. We noted a case (WA_24) of intrapatient, intralesional heterogeneity with early metastatic spread and a spectrum of NE differentiation. All sites of WA_24 were clonally related but did not share truncal mutations, indicating that the cancer, driven by a truncal *TMPRSS2–ERG* fusion, clonally diverged and disseminated prior to the acquisition of exonic mutations. This early phylogenetic branching is further reflected in profound transcriptional and histologic heterogeneity. Woodcock and colleagues (65) have reported a similar case (A34) wherein two early branching clones independently acquired metastatic potential. However, in that patient, the tumor had acquired multiple mutations before early dissemination. We also identified examples (WA_22 and WA_32) of recurrent seeding of distal metastases from the prostate. Ongoing clonal evolution within the unresected prostate resulted in multiple waves of dissemination and reseeding, suggesting an active role for prostate tumors in fueling metastatic disease. This finding along with studies showing that prostate radiotherapy in subsets of men with metastatic prostate cancer improves failure-free and overall survival (70) highlights the importance of considering primary therapy in patients with metastatic prostate cancer. Together, these observations support the existence of multiple, but always related, clones within primary prostate cancer with metastatic potential (4).

The current study has several limitations. Although using WES data, as opposed to WGS, allows for higher sequencing depths and increases the sensitivity of detecting variants with low CCF, it detects fewer mutations, which may result in the failure of detecting some clones. In this cohort, we were unable to procure and sequence all metastatic lesions from a patient and oversampled those from the liver and other tissues relative to bone. This has both advantages, in terms of statistical power and comprehensive characterization of lesions associated with poor outcomes, and disadvantages, i.e., deviation from the typical presentation of mCRPC. Increasing the number of sequenced samples per patient would further increase the ability to characterize patterns of metastatic dissemination and possibly improve analyses. Some of our results are based on mathematical modeling and use of inferential algorithms. Although we cross-reference these results with descriptive data, inferences of processes that cannot be directly observed (e.g., tumor evolution) are always associated with uncertainty.

In summary, our study highlights the profound genetic heterogeneity of lethal prostate cancer and examines the critical contribution of early dissemination and independent evolution at metastatic sites to its complex clonal architecture. We demonstrate that except for a few truncal drivers, sampling multiple sites increases diagnostic yields both at the patient and cohort levels. Lastly, the majority of patients in our cohort received a uniform treatment regimen, which limited our ability to assess the impact of different treatments on mCRPC evolution and genomic heterogeneity. Further studies are required to characterize the effect of different treatment regimens on tumor heterogeneity.

## Supplementary Material

Figure S1 Supplementary Figure 1: Overview of the cohort and ubiquitous alterations across metastatic tumors in mCRPC (supplement)

Figure S2 Supplementary Figure 2: Mutational heterogeneity in mCRPC and its impact on detecting alterations using a single sample per patient (supplement)

Figure S3 Supplementary Figure 3: Heterogeneity of recurrent copy number alterations across metastatic sites (supplement)

Figure S4 Supplementary Figure 4: Patterns of clonal evolution and metastatic dissemination in mCRPC (supplement)

Table S1 Supplementary Table 1: Results of the DeTiN indicating tumor in normal contamination

Table S2 Supplementary Table 2: Corresponding site for normal and tumor samples

Table S3 Supplementary Table 3: Alteration frequencies

Table S4 Supplementary Table 4: Corresponding purity, ploidy and WGD of tumors

Table S5 Supplementary Table 5: Tumor RNA counts

Table S6 Supplementary Table 6: Matched normal RNA counts

Table S7 Supplementary Table 7: Patients treatment history

Table S8 Supplementary Table 8: QC measures for DNA samples

Table S9 Supplementary Table 9: QC measures for RNA samples

Table S10 Supplementary Table 10: List of mutation calls

Table S11 Supplementary Table 11: List of copy number calls

Table S12 Supplementary Table 12: Mutations and their inferred CCF clusters

Table S13 Supplementary Table 13: List of WA_24 segments with LOH

## Data Availability

All processed data required for the reproduction of the results are included as part of Supplementary Tables S1–S13. Raw sequencing data are available under accession number SRA PRJNA1256916. All other raw data are available upon request to the corresponding author.
