# Supplementary material for: Divergent Clonal Evolution and Early Dissemination Promote Genetic Heterogeneity of Metastases in Castration-Resistant Prostate Cancer
Source: Cancer Res. 2025 Aug 18;85(21):4251–68. doi: 10.1158/0008-5472.CAN-24-3687 (PMC12580794; doi:10.1158/0008-5472.CAN-24-3687)
Supplement: Figure S1 — Supplementary Figure 1: Overview of the cohort and ubiquitous alterations across metastatic tumors in mCRPC (supplement) [file can-24-3687_figure_s1_suppsf1.pdf]

Figure S1

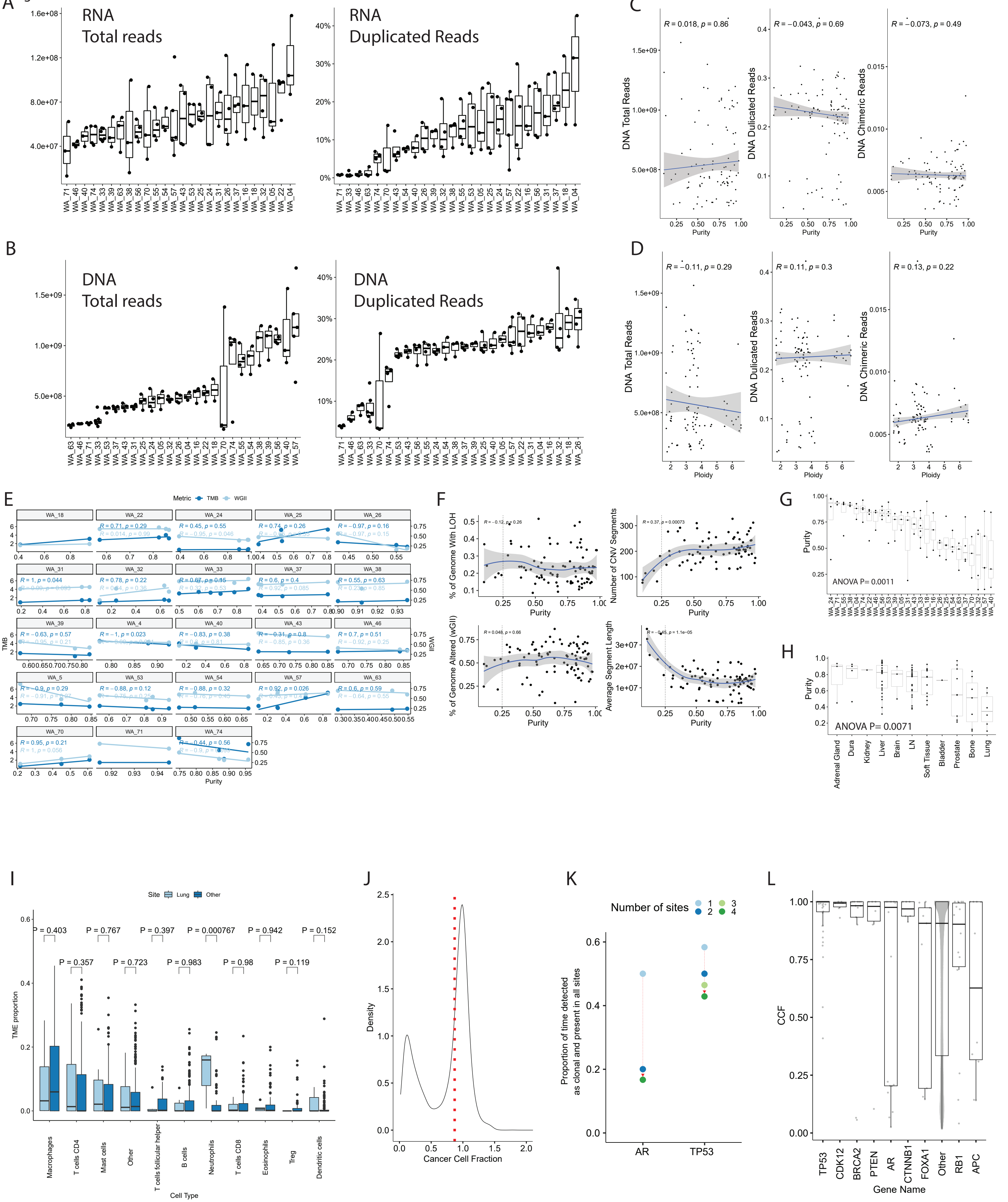
A
RNA
Total reads
RNA
Duplicated Reads
B
DNA
Total reads
DNA
Duplicated Reads
C
R = 0.018, p = 0.86
R = −0.043, p = 0.69
R = −0.073, p = 0.49
DNA Total Reads
DNA Dulicated Reads
DNA Chimeric Reads
Purity
D
R = −0.11, p = 0.29
R = 0.11, p = 0.3
R = 0.13, p = 0.22
Ploidy
E
Metric
TMB
WGII
Purity
F
% of Genome With LOH
Number of CNV Segments
% of Genome Altered (wGII)
Average Segment Length
Purity
G
Purity
ANOVA P= 0.0011
H
Purity
ANOVA P= 0.0071
Adrenal Gland
Dura
Kidney
Liver
Brain
LN
Soft Tissue
Bladder
Prostate
Bone
Lung
I
Site
Lung
Other
TME proportion
P = 0.403
P = 0.357
P = 0.767
P = 0.723
P = 0.397
P = 0.983
P = 0.000767
P = 0.98
P = 0.942
P = 0.119
P = 0.152
Macrophages
T cells CD4
Mast cells
Other
T cells follicular helper
B cells
Neutrophils
T cells CD8
Eosinophils
Treg
Dendritic cells
Cell Type
J
Density
Cancer Cell Fraction
K
Number of sites
1
2
3
4
Proportion of time detected as clonal and present in all sites
AR
TP53
L
CCF
TP53
CDK12
BRCA2
PTEN
AR
CTNNB1
FOXA1
Other
RB1
APC
Gene Name

Supplementary Figure 1: Overview of the cohort and ubiquitous alterations across metastatic tumors in mCRPC (supplement)

(A) QC measures for RNA data. The left panel shows the total number of reads for each sample. The middle panel shows the proportion of duplicated reads and the right panel shows the proportion of ribosomal RNA in each sample.

(B) QC measures for DNA data. The left panel shows the total number of reads for each sample. The middle panel shows the proportion of PCR duplicated reads and the right panel shows the proportion of chimeric reads in each sample.

(C, D) Association between DNA QC measures and inferred purity (C) and ploidy (D) for each sample, indicating that sample quality did not significantly impact the downstream inferences of purity or ploidy.

(E) Association between purity and the TMB (top) and wGII (bottom). The plot indicates that purity is not significantly affecting the ability to call mutations and CNVs in this cohort.

(F) Association between purity and CNV related measures, indicating the effect of purity in lower sensitivity for samples with purity < 25%

(G, H) Purity (based on NGS) of tumors stratified by patient (F=3.49, Degree of freedom=25) (G) or tissue (F=3.19, Degree of freedom=10) (H). Each dot represents one tumor. ANOVA P-value indicates that purity is significantly associated with the patient (G) or tissue (H) from which the tumor is obtained.

(I) Proportion of each cell type in the TME. Light blue indicates proportion of each cell type in lung metastasis and dark blue indicates proportions for all other primary and metastatic sites.

(J) Density distribution of CCF values for all the non-synonymous mutations. Red line indicates the threshold used to call clonal versus subclonal mutations

(K) Average proportion of *AR* and *TP53* mutations detected as present and clonal in all the evaluated sites. Only patients with at least 4 sequenced tumors are included.

(L) CCF of mutations in genes recurrently altered in PCa compared to all other mutations.
