# Supplementary material for: Divergent Clonal Evolution and Early Dissemination Promote Genetic Heterogeneity of Metastases in Castration-Resistant Prostate Cancer
Source: Cancer Res. 2025 Aug 18;85(21):4251–68. doi: 10.1158/0008-5472.CAN-24-3687 (PMC12580794; doi:10.1158/0008-5472.CAN-24-3687)
Supplement: Figure S2 — Supplementary Figure 2: Mutational heterogeneity in mCRPC and its impact on detecting alterations using a single sample per patient (supplement) [file can-24-3687_figure_s2_suppsf2.pdf]

Figure S2

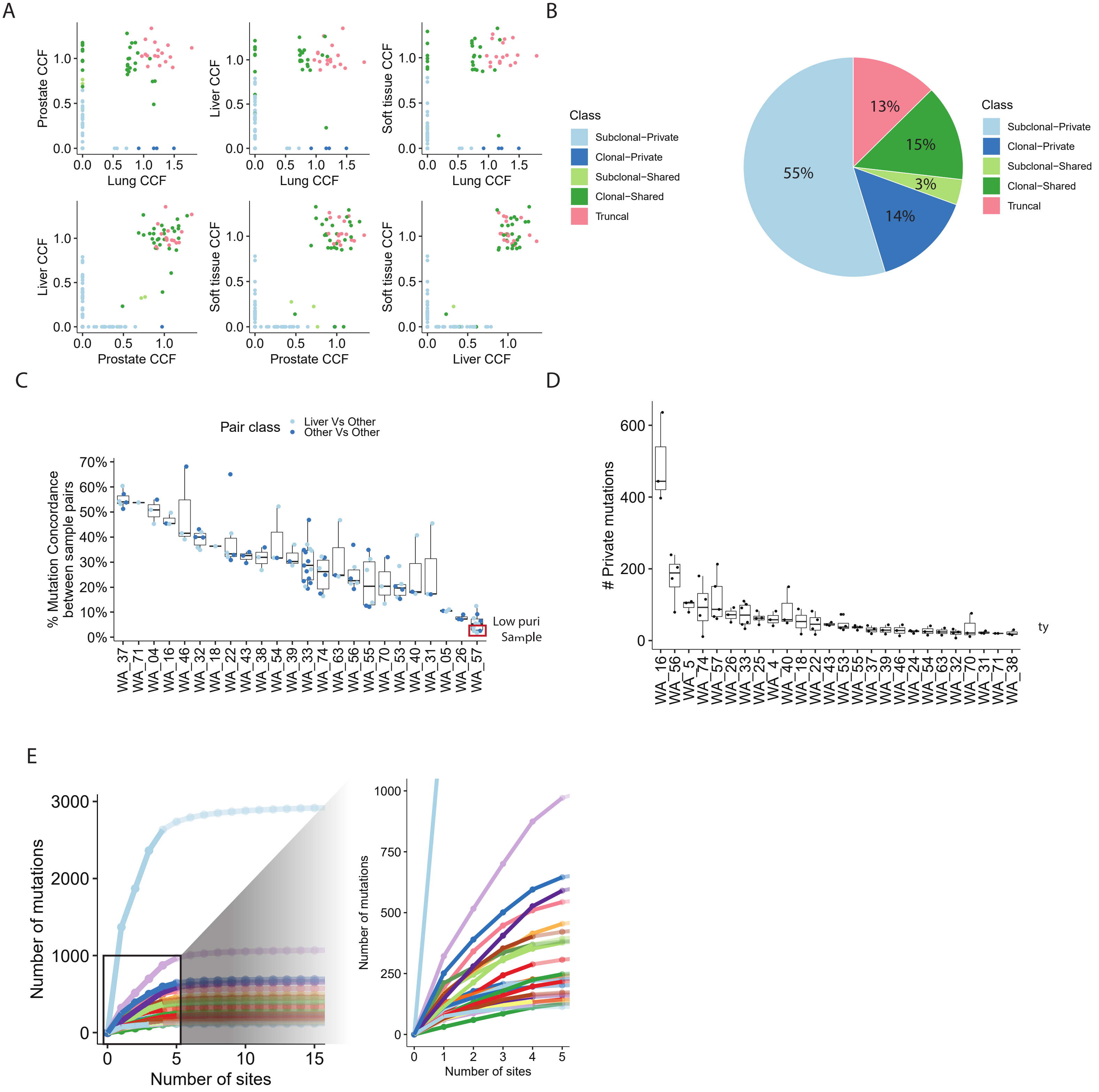

A
Prostate CCF
Lung CCF
Liver CCF
Soft tissue CCF
Prostate CCF
Liver CCF
0.0
0.5
1.0
1.5
Class
Subclonal−Private
Clonal−Private
Subclonal−Shared
Clonal−Shared
Truncal
B
13%
15%
3%
14%
55%
Class
Subclonal−Private
Clonal−Private
Subclonal−Shared
Clonal−Shared
Truncal
C
Pair class
Liver Vs Other
Other Vs Other
% Mutation Concordance between sample pairs
70%
60%
50%
40%
30%
20%
10%
0%
Low puri
Sample
WA_37
WA_71
WA_04
WA_16
WA_46
WA_32
WA_18
WA_22
WA_43
WA_38
WA_54
WA_39
WA_33
WA_74
WA_63
WA_56
WA_55
WA_70
WA_53
WA_40
WA_31
WA_05
WA_26
WA_57
D
Private mutations
600
400
200
0
ty
WA_16
WA_56
WA_5
WA_74
WA_57
WA_26
WA_33
WA_25
WA_4
WA_40
WA_18
WA_22
WA_43
WA_53
WA_55
WA_37
WA_39
WA_46
WA_24
WA_54
WA_63
WA_32
WA_70
WA_31
WA_71
WA_38
E
Number of mutations
3000
2000
1000
0
Number of sites
0
5
10
15
Number of mutations
1000
750
500
250
0
Number of sites
0
1
2
3
4
5

Supplementary Figure 2: Mutational heterogeneity in mCRPC and it's impact on detecting alterations using a single sample per patient (supplement)

(A) Illustration of mutation classification for one patient (WA_32). Private mutations appear always on the edge of all plots, while Truncal mutations are always in the middle. Shared and subclonal mutations have variable positions.

(B) Proportion of each mutation class across all patients in this cohort

(C) Concordance of mutations for all pairs of samples from each patient. Each y-axis value represents the proportion of mutations detected in both of a pair of samples (i.e., concordance). For a patient with n sites, there are n choose 2 pairs.

(D) Number of private mutations stratified by patient. Each dot represents the number of private mutations in one tumor.

(E) Zoomed version of panel (J) in Figure 2
