# Supplementary material for: Divergent Clonal Evolution and Early Dissemination Promote Genetic Heterogeneity of Metastases in Castration-Resistant Prostate Cancer
Source: Cancer Res. 2025 Aug 18;85(21):4251–68. doi: 10.1158/0008-5472.CAN-24-3687 (PMC12580794; doi:10.1158/0008-5472.CAN-24-3687)
Supplement: Figure S3 — Supplementary Figure 3: Heterogeneity of recurrent copy number alterations across metastatic sites (supplement) [file can-24-3687_figure_s3_suppsf3.pdf]

Figure S3

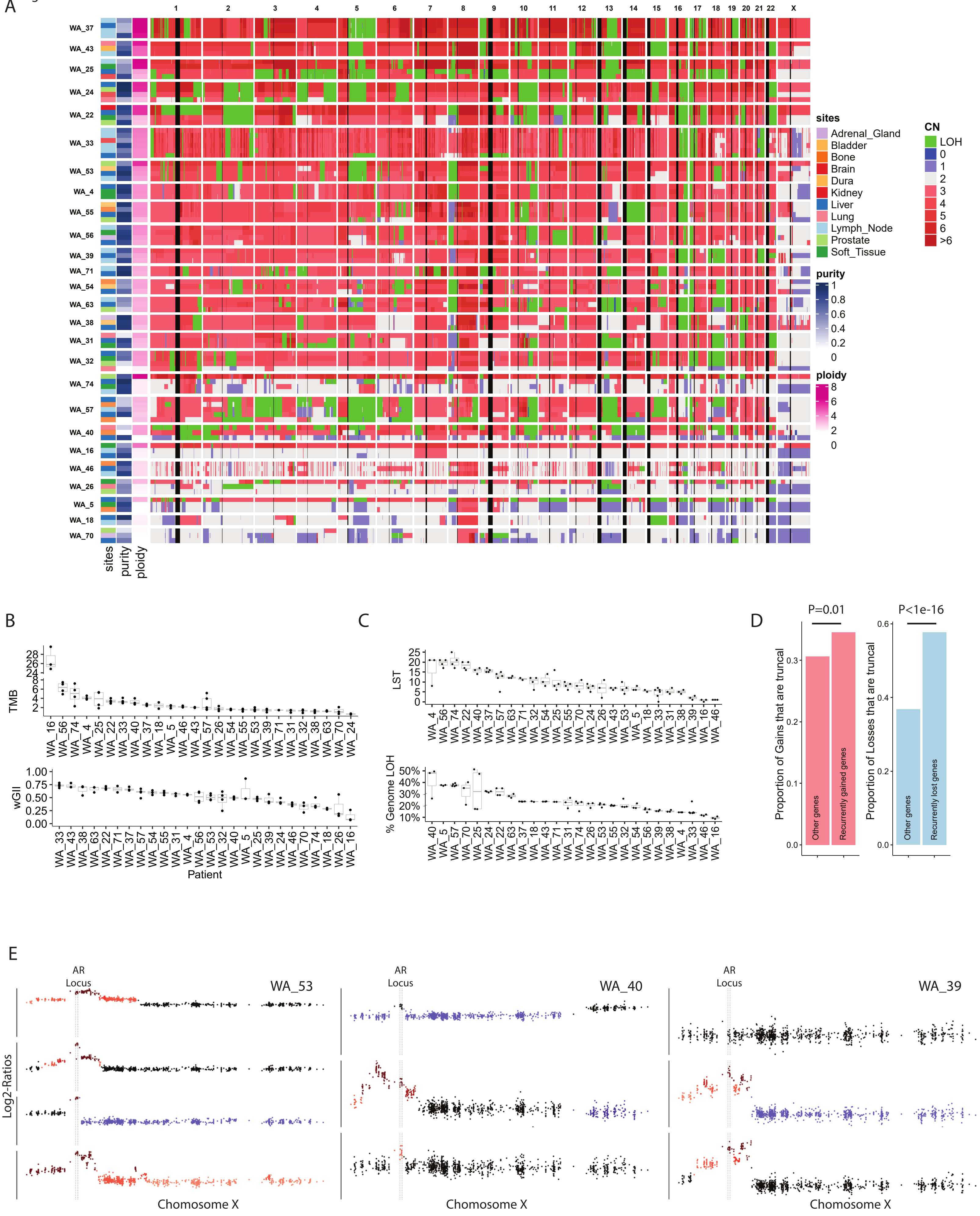
A
1
2
3
4
5
6
7
8
9
10
11
12
13
14
15
16
17
18
19
20
21
22
X
WA_37
WA_43
WA_25
WA_24
WA_22
WA_33
WA_53
WA_4
WA_55
WA_56
WA_39
WA_71
WA_54
WA_63
WA_38
WA_31
WA_32
WA_74
WA_57
WA_40
WA_16
WA_46
WA_26
WA_5
WA_18
WA_70
sites
purity
ploidy
sites
Adrenal_Gland
Bladder
Bone
Brain
Dura
Kidney
Liver
Lung
Lymph_Node
Prostate
Soft_Tissue
CN
LOH
0
1
2
3
4
5
6
>6
purity
1
0.8
0.6
0.4
0.2
0
ploidy
8
6
4
2
0
B
TMB
Patient
wGII
C
LST
% Genome LOH
D
P=0.01
P<1e-16
Proportion of Gains that are truncal
Proportion of Losses that are truncal
Other genes
Recurrently gained genes
Other genes
Recurrently lost genes
E
AR
Locus
WA_53
WA_40
WA_39
Log2-Ratios
Chomosome X
Chomosome X
Chomosome X

Supplementary Figure 3: Heterogeneity of recurrent copy number alterations across metastatic sites (supplement)

(A) Per patient and site genome-wide copy number alteration landscape. Colors indicate copy-number alterations (red - gain, blue - loss, green - LOH, gray - diploid). Cases and samples ordered by average ploidy.

(B) Heterogeneity of the weighted genome instability index (wGII) and TMB across metastatic sites.

(C) Heterogeneity of homologous recombination deficiency (HRD) scores derived from CNV data. Large-scale state transitions (LST) scores (top), and proportion of genome harboring LOH (bottom).

(D) Proportion of recurrently truncal gains (left) or losses (right) within regions of the genome stratified by alteration recurrence (Methods).

(E) AR locus visualization of normalized log2(tumor / normal) coverage ratios across samples from three index cases (black - diploid, red - gain, blue - loss).
