# Supplementary material for: Divergent Clonal Evolution and Early Dissemination Promote Genetic Heterogeneity of Metastases in Castration-Resistant Prostate Cancer
Source: Cancer Res. 2025 Aug 18;85(21):4251–68. doi: 10.1158/0008-5472.CAN-24-3687 (PMC12580794; doi:10.1158/0008-5472.CAN-24-3687)
Supplement: Figure S4 — Supplementary Figure 4: Patterns of clonal evolution and metastatic dissemination in mCRPC (supplement) [file can-24-3687_figure_s4_suppsf4.pdf]

Figure S4

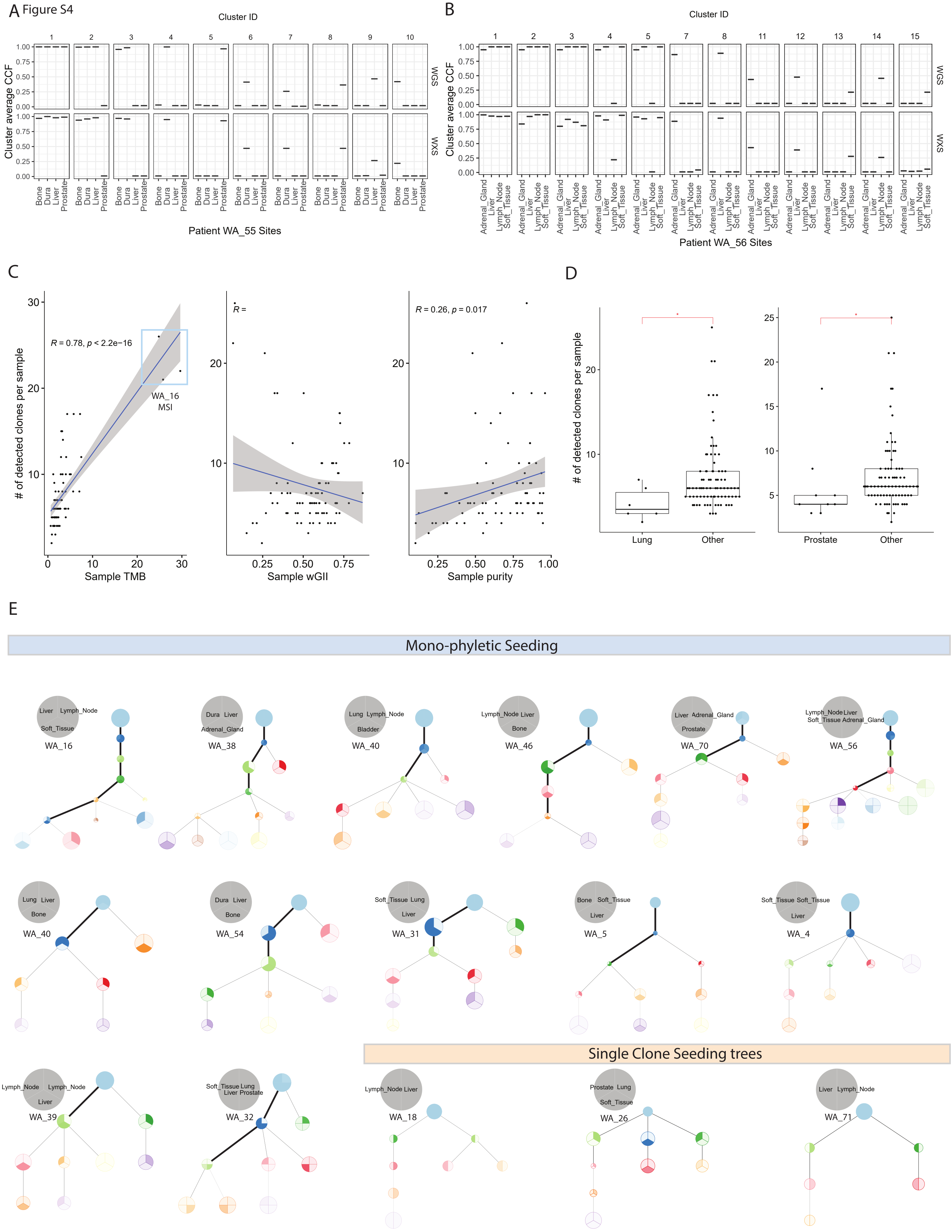
A
Cluster ID
1 2 3 4 5 6 7 8 9 10
Cluster average CCF
WGS
WXS
Bone
Dura
Liver
Prostate
Patient WA_55 Sites
B
Cluster ID
1 2 3 4 5 7 8 11 12 13 14 15
Cluster average CCF
WGS
WXS
Adrenal_Gland
Liver
Lymph_Node
Soft_Tissue
Patient WA_56 Sites
C
# of detected clones per sample
R = 0.78, p < 2.2e−16
WA_16
MSI
Sample TMB
R =
Sample wGII
R = 0.26, p = 0.017
Sample purity
D
# of detected clones per sample
Lung
Other
Prostate
Other
E
Mono-phyletic Seeding
WA_16
WA_38
WA_40
WA_46
WA_70
WA_56
WA_40
WA_54
WA_31
WA_5
WA_4
WA_39
WA_32
Single Clone Seeding trees
WA_18
WA_26
WA_71

Supplementary Figure 4: Patterns of clonal evolution and metastatic dissemination in mCRPC (supplement)

(A) Cluster average CCF for patient WA_55 per each site. Top row indicates the clusters detected in WGS and bottom row indicate clusters detected in WES. Clusters with matching average CCF profiles are in each column to show the similarity of detected clusters in each approach

(B) Cluster average CCF for patient WA_56 per each site. Top row indicates the clusters detected in WGS and bottom row indicate clusters detected in WES. Clusters with matching average CCF profiles are in each column to show the similarity of detected clusters in each approach

(C) Number of clones detected in each sample stratified by TMB, wGII and purity of the samples. Significance based on Pearson correlation test.

(D) Number of clones detected in each sample stratified by tissue site. Significance based on Wilcoxon rank sum test.

(E) Classification of clonal phylogenetic trees based on ancestral relationships between seeding clones. See (Figure 4E,F) for details. Single-clone seeding represents trees where all metastatic sites were seeded by the same clone.
